# Dual-locked targeted alpha-emitter enhanced tumor immunotherapy via Diels–Alder reaction-based self-immolative molecular cage strategy

**DOI:** 10.1186/s40779-025-00673-5

**Published:** 2025-12-01

**Authors:** Meng-Die Yang, Kang Fang, Xiao-Yi Zhang, Gang Yang, He-Qing Yi, Le Cai, Shan-Shan Qin, Xin-Da Yang, Rong Rong, Shuo Shi, Fei Yu

**Affiliations:** 1 Department of Nuclear Medicine, Shanghai Tenth People’s Hospital, Tongji University School of Medicine, School of Chemical Science and Engineering, Shanghai, 200072 China; 2 Institute of Nuclear Medicine, Tongji University School of Medicine, Shanghai, 200072 China; 3 College of Pharmacy, Shandong University of Traditional Chinese Medicine, Jinan, 250355 China; 4 Department of Nuclear Medicine, Zhejiang Cancer Hospital, Hangzhou, 310022 China

**Keywords:** Targeted alpha therapy (TAT), Alpha-emitters, Radium-223 (^223^Ra), Bioorthogonal click chemistry

## Abstract

**Background:**

Targeted alpha therapy (TAT) has emerged as a promising strategy for cancer treatment by selectively delivering high linear energy transfer (LET) alpha-emitters to tumor cells while minimizing off-target toxicity. However, the clinical translation of alpha-emitters, particularly radium-223 (^223^Ra), remains challenging due to inefficient targeted delivery and uncontrolled release of recoil daughter products, leading to systemic toxicity.

**Methods:**

Herein, a dual-locked pretargeted strategy was developed integrating platinum^IV^ (Pt^IV^)-loaded hydrogel nanoparticles (HNPs) (HAQ@HNPs) and ^223^Ra-loaded HNPs (^223^Ra@HNPs) into an inverse electron demand Diels–Alder (IEDDA)-activated drug delivery system. In vitro cytotoxicity, ROS, and apoptosis, together with in vivo biodistribution, imaging, and therapeutic studies, were performed to evaluate the therapeutic efficacy and immune activation.

**Results:**

This caged dual-locked approach enables precise pretargeted accumulation at the tumor site, followed by rapid dissociation and controlled release of ^223^Ra and Pt^IV^ upon IEDDA-triggered activation, thereby ensuring high tumor specificity while minimizing systemic exposure. The synergistic combination of TAT and chemotherapy effectively disrupts redox homeostasis, induces immunogenic cell death (ICD), and elicits a robust antitumor immune response. Furthermore, when combined with programmed death-ligand 1 (PD-L1) blockade, this strategy significantly enhances systemic antitumor immunity, leading to robust inhibition of tumor growth and metastasis.

**Conclusions:**

These findings underscore the potential of dual-locked pretargeted strategies to advance TAT by improving therapeutic efficacy and addressing the critical challenge of radionuclide leakage, paving the way for next-generation precision-targeted radiopharmaceuticals.

**Supplementary Information:**

The online version contains supplementary material available at 10.1186/s40779-025-00673-5.

## Background

Targeted alpha therapy (TAT), which has unique advantages and is unaffected by the resistance typically seen in the majority of other tumor treatments, has gained popularity in recent years [1]. Specifically, alpha-particles are helium nuclei composed of 2 protons and 2 charged neutrons, with a high linear energy transfer (LET) of 50–230 keV/μm [2] and a high relative biological effectiveness (RBE) [3]. Moreover, alpha-particles can limit excessive radiation exposure because of their very short distances (50–100 µm), which allows them to eliminate only the cancer cells while preserving as much healthy tissues as possible [4]. It is worth mentioning that radium-223 chloride (^223^RaCl_2_, Xofigo^®^, Bayer, Germany) was approved in 2013 as the first alpha-particle radiopharmaceutical for clinical use. The physical half-life of ^223^Ra is 11.4 d, and its decay emits high-energy alpha-particles with a short range (< 100 μm), which has great clinical application potential [5]. ^223^Ra, due to its osteophilic characteristics, is often used to treat patients with metastatic castration-resistant prostate cancer (mCRPC) [6]. However, radium, as an alkaline earth metal, poses challenges for chelation reactions of Ra^2+^ in aqueous solutions [7]. The development of novel radiopharmaceuticals that can target other non-osseous solid tumors is still hampered by the enormous difficulty of labeling biological carriers with ^223^Ra.

To stabilize anchor ^223^Ra for precise tumor targeting, various liposomes, polymers, or particles based on metals or inorganic substances, such as zeolites [8, 9], hydroxyapatite [10–12], titanium dioxide (TiO_2_) [13], alpha-zirconium phosphate (α-ZrP) [14], superparamagnetic iron-oxide (SPION) [15], single-atom nanozymes [16], and layered double hydroxide (LDH) [17], have been studied for use in nuclear medicine. In addition to the inherent difficulty of fixing ^223^Ra within carriers, its long decay chain generates four daughter radionuclides, radon-219 (^219^Rn), polonium-215 (^215^Po), lead-211 (^211^Pb), and thallium-207 (^207^Th), that possess high recoil energies. These recoil events can break chemical bonds and lead to the redistribution of radioactive daughters within the body [18]. Therefore, the development of innovative radiopharmaceuticals based on ^223^Ra require achieving precise tumor-targeting capabilities and reducing the leakage of daughter radionuclides. Recent studies pointed out that nanoparticles provided precise tumor targeting, improved radionuclide retention as well as resistance to daughter recoils redistribution, when compared to small molecules [19, 20]. Among radionuclide nanoparticle-carriers, hydrogel stood out due to their unique water solubility, excellent biocompatibility, and tumor retention [21]. Besides, it is believed that combining active targeting techniques with the enhanced permeability and retention (EPR) effect of nanomaterials, which are capable of precisely identifying and attaching to tumor cells, is essential to improving the accuracy and therapeutic efficacy of ^223^Ra-based radiopharmaceuticals [22, 23]. Therefore, ^223^Ra carrier platforms with enhanced targeting specificity and improved radionuclide encapsulation, developed under this strategy, should be actively pursued.

Fortunately, a novel tumor treatment strategy termed “dual-locked” served as the conceptual basis for our therapeutic design, which could only be unlocked to activate therapy based on dynamic covalent linkages. This strategy requires 2 distinct triggers, typically biological recognition and chemical activation, for therapeutic release, which greatly reduces premature leakage and systemic side effects [24, 25]. However, the development of dual-locked strategy was relatively lacking in nuclide delivery on cancer treatment, particularly with alpha-emitting isotopes. It was an attractive direction to develop a dual-locked strategy toward nuclide delivery to enhance the tumor accumulation and induce damage to normal tissues. Notably, pretargeted strategy based on the inverse electron demand Diels–Alder (IEDDA) reaction has been shown to enhance radiopharmaceuticals uptake in tumors by achieving in situ self-assembly [26, 27]. However, enhancing radionuclide accumulation at tumor sites by intelligent control of radionuclide release presents significant challenge. Adoption of an ideal stimuli-responsive building block would be the key to achieving this goal. To address this, we proposed that the 2-hydroxy-5-methyl-1,3-phenylenedimethanol motif as a building block could engineer the benzylic positions symmetrically with drug/targeting moieties through carbonate linkages [28]. Under this design, the caged drug/targeting moieties could be strictly released simultaneously in a 1:1 mode through self-immolation procedures based on elimination by an electronic cascade, meanwhile with much less substrate-dependent kinetics. Once the cyclization of a molecule (1,4-electron elimination self-immolation procedures) was activated by the IEDDA reaction, the caged-molecular fragment would be released, resulting in cleavage (self-immolation) [29]. On the basis of this concept, selecting an appropriate polymer molecule as hydrogel coating could be used to protect the radionuclide from premature leakage in the blood circulation. Polyethylene glycol (PEG) has attracted our attention due to its excellent properties such as biodegradability, biocompatibility, and encapsulation properties [30]. This caged dual-locked strategy provided a promising perspective for enhancing the tumor targeting of radionuclides and reducing leakage.

Clinically, the broader application of ^223^Ra remains limited by its chemical inertness as an alkaline earth metal, which complicates stable coordination chemistry, and by the lack of efficient delivery systems capable of targeting non-osseous tumors. To address these challenges, we designed an IEDDA reaction-based dual-locked pretargeting system for ^223^Ra, enabling precise tumor accumulation and minimizing radionuclide leakage.

## Materials and methods

### Synthesis of oxaliplatin prodrug [platinum^IV^ (Pt^IV^)]

The mixture contained oxaliplatin and 100 ml of 30% hydrogen peroxide (H_2_O_2_) was stirred at 60 ℃ over 6 h, then the clear solution was freeze-dried to obtain white powder. Put the white powder into dimethyl sulfoxide (DMSO) containing excessive succinic anhydride. The mixture was allowed to react at room temperature for 12 h, then Pt^IV^ as white powder was obtained by freeze-dried the clear solution [31].

### Synthesis of self-immolating molecule

Biotin-PEG-NH_2_ (average molecular weight = 1000 Da) and triethylamine (TEA) were injected into compound 7 (the synthesis of compound 7, details in Additional file 1: Methods) in anhydrous N,N-dimethylformamide (DMF) using a syringe over a period of 15 min. The resulting mixture was allowed to equilibrate to room temperature and maintained for 12 h. The clear solution was freeze-dried to remove solvent. Subsequently, the reaction mixture was diluted with ethyl acetate, and purified by column chromatography (CH_2_Cl_2_:CH_3_OH from 100:1 to 10:1) to collect red mobile phase to yield self-immolating molecule.

### Synthesis of Pt^IV^-loaded hydrogel nanoparticles (HNPs) with HAQ [hyaluronic acid (HA) with bicyclo[6.1.0]nonyne (BCN)] (HAQ@HNPs)

The blank nanohydrogels with carboxyl groups (HNPs-COOH) were synthesized according to previously reported methods [32]. Span 80 and Tween 80 were dissolved in cyclohexane, followed by the addition of a mixed solution containing poly (ethylene glycol) diacrylate (PEGDA), water, and 2-carboxyethyl acrylate. The resulting mixture was transformed into an emulsion solution through ultrasonication for 2 min. Subsequently, the photoinitiator 2-hydroxy-2-methylpropiophenone was incorporated into the emulsion. The reaction was carried out under magnetic stirring at 800 rpm using a 365 nm longwave ultraviolet (UV) lamp for 3 h, all while being protected by nitrogen. The blank HNPs were collected via centrifugation at 12,000 rpm and washed 3 times with ethanol and water. Pt@HNPs were prepared by mixing Pt^IV^ and blank HNPs for 4 h under dark conditions, and collected by centrifugation at 12,000 rpm and washed 3 times with water, then the nanoparticle solution was freeze-dried to obtain dry Pt@HNPs. N-hydroxysuccinimide (NHS), 1-ethyl-3-(3-dimethylaminopropyl) carbodiimide (EDC), 4-dimethylaminopyridine (DMAP; catalytic amount), and dry Pt@HNPs were dissolved in DMF. The mixture was stirred at room temperature for 15 min under argon protection. HAQ (2 mg) was then added and the resulting solution was stirred overnight at room temperature under argon protection. The HAQ@HNPs were collected by centrifugation (12,000 rpm) and washed 3 times with ethanol and water.

### Synthesis of Ba@HNPs

Firstly, the blank nanohydrogels with amino groups (HNPs-NH_2_) were synthesized referring to the synthetic method of HNPs-COOH. The difference between the two approaches is that 2-carboxyethyl acrylate was replaced by 1-amino-3-butene hydrochloride. Ba-HNPs were prepared by mixing Ba^2+^ and blank HNPs for 4 h under dark conditions, and collected by centrifugation at 12,000 rpm and washed 3 times with water. NHS, EDC, and Ba-HNPs were dissolved in H_2_O. The mixture was stirred at room temperature for 30 min. Self-immolating molecule (2 mg) was then added, and the resulting solution was stirred overnight at room temperature under argon protection. The Ba@HNPs were collected by centrifugation (12,000 rpm) and washed 3 times with ethanol and water.

### Synthesis of ^223^Ra-loaded HNPs (^223^Ra@HNPs)

The synthesis procedure is comparable to that of Ba@HNPs, with the exception that ^223^Ra^2+^ is produced using a ^223^RaCl_2_ solution. The stability of ^223^Ra@HNPs was evaluated using paper chromatography on Whatman No. 1 filter paper. Samples were incubated in phosphate-buffered saline (PBS) or 10% fetal bovine serum (FBS) at 37 °C for varying durations (0, 6, 12, and 24 h), followed by radioactivity measurement using a γ-counter.

### In vitro cytotoxicity assay

B16/F10 cells, Lewis lung carcinoma (LLC) cells, or human umbilical vein endothelial cells (HUVECs) were obtained from the Cell Bank of the Chinese Academy of Sciences (Shanghai, China). Different cells (5 × 10^3^ cells/well) were seeded in 96-well plates and incubated with varying concentrations of Pt^IV^, free ^223^Ra, HAQ@HNPs, ^223^Ra@HNPs, and HAQ/^223^Ra@HNPs (Pt^IV^-loaded HNPs with HAQ and ^223^Ra-loaded HNPs with self-immolating molecule) for 24 h. The drug concentrations were adjusted to maintain a consistent Pt^IV^ or ^223^Ra content across relevant groups. After incubation, cell counting kit-8 (CCK-8) solution was added, and absorbance at 450 nm was measured using a microplate reader to assess cell viability.

### Endoplasmic reticulum (ER) morphology analysis

B16/F10 cells were treated with the same conditions as above for 24 h. Cell samples were initially fixed in 2.5% glutaraldehyde and subsequently post-fixed in buffered 1% osmium tetroxide. They were then dehydrated through a graded series of acetone and embedded in Epon resin. Ninety-nanometer-thin sections were cut using a Leica EM UC6 ultramicrotome (Leica Microsystems, Wetzlar, Germany). The sections were stained with a saturated solution of uranyl acetate and lead citrate. Images were captured using a Hitachi transmission electron microscopy (TEM) system (Hitachi High-Technologies Corporation, Tokyo, Japan).

### Animal tumor model

Female C57BL/6 mice (4–5 weeks old, *n* = 175) were purchased from Shanghai Jihui Laboratory Animal Breeding Co., Ltd. (Shanghai, China; license No. SCXK[Hu]2022-0009) and housed under standard laboratory conditions. All animal procedures were approved by the Institutional Animal Care and Use Committee of Shanghai Tenth People’s Hospital (SHDSYY-2023-0746).

### Biodistribution, dosimetry, and digital autoradiography (DAR) study

Tumor-bearing C57BL/6 mice received an intra-tumoral injection when the tumor diameter reached approximately 100 mm^3^. As for biodistribution study of free ^223^Ra, ^223^Ra@HNPs, and HAQ/^223^Ra@HNPs, organs including tumor, heart, liver, spleen, lungs, kidneys, stomach, large intestines, small intestines, bone, muscle, brain, blood, and tail were extracted to weigh and quantified radioactivity using a γ-counter, the corresponding radioactivity concentrations (%ID/g) were calculated. When calculating tumor-to-organ uptake ratios, another panel of mice models were conducted at multiple time points (2, 6, 12, 24, 48, and 72 h) following free ^223^Ra and HAQ/^223^Ra@HNPs injection, covering organs including tumor, heart, liver, spleen, lungs, kidneys, small intestines, and bone. Based on these corresponding radioactivity concentrations, these data were fitted using MATLAB (R2022b, MathWorks, Natick, MA, USA) to generate time-activity curves, from which the area under the curve was calculated to estimate the time-integrated activity (TIA). Absorbed dose was then estimated using the medical internal radiation dose (MIRD) formalism via the MIRDcalc software (https://mirdsoft.org/mirdcalc). As for DAR study, at 72 h post-administration, the tumor, kidney, and liver tissues were harvested and immediately embedded in optimal cutting temperature (OCT) compound, snap-frozen in liquid nitrogen, and sectioned at 8–10 μm thickness using a cryostat. The frozen sections were then subjected to DAR to assess the intratissue distribution of radioactivity.

### In vivo antitumor study and immune response analysis

B16/F10 cells or LLC cells (1 × 10^6^ cells per dish) were subcutaneously injected into the right flanks of C57BL/6 mice. When tumors reached 50–100 mm^3^, mice were randomly divided into 6 groups: control, Pt^IV^, free ^223^Ra, HAQ@HNPs, ^223^Ra@HNPs, and HAQ/^223^Ra@HNPs. Tumor size and body weight were measured every 2 d. Tumor volume was calculated as: volume = width^2^ × length/2. After 10 d of treatment, mice were euthanized, and major organs (heart, liver, spleen, lungs, and kidneys) were collected for histopathological examination using hematoxylin and eosin (H&E) staining. Blood samples were analyzed for liver and kidney function markers and complete blood count parameters to assess systemic toxicity.

For immune response analysis, after 7 days of treatment, tumors were harvested and processed into single-cell suspensions. Immune cell populations were analyzed by flow cytometry (FCM) using fluorescently labeled antibodies targeting CD45, CD3, CD4, CD8, forkhead box P3 (FOXP3), CD11c, CD80, and CD86. Data were acquired via FCM and analyzed using FlowJo software (FlowJo LLC, BD Biosciences, Ashland, OR, USA).

### In vivo combination therapy in a bilateral tumor model

To evaluate systemic antitumor effects, B16/F10 cells were inoculated bilaterally (1 × 10^6^ cells in the left flank) in C57BL/6 mice. When the tumor size reached 50–100 mm^3^ (after 7 d), the mice were randomly grouped for treatment, including 8 groups [control, anti programmed death-ligand 1 (PD-L1), HAQ@HNPs, HAQ@HNPs + anti PD-L1, ^223^Ra@HNPs, ^223^Ra@HNPs + anti PD-L1, HAQ/^223^Ra@HNPs, and HAQ/^223^Ra@HNPs + anti PD-L1]. Treatments were administered on day 0 (or days 0, 2, 4), and then additional B16/F10 cells (1 × 10^6^ cells) were implanted in the right flank to simulate metastatic lesions on day 8. Tumor growth and body weight were monitored every other day. Single-cell suspensions from distant tumors and spleens were analyzed for memory and activated T cells via FCM.

### Statistical analysis

Statistical tests were performed using GraphPad Prism 9. Data are presented as mean ± standard deviation (SD). One-way or two-way ANOVA followed by Tukey’s or Bonferroni’s post hoc test was used for pairwise comparisons, or Student’s *t*-test was applied for two-group analyses. *P* < 0.05 was considered to indicate statistical significance.

## Results

### Preparation and characterization of a pretargeted delivery system (HAQ@HNPs)

**Fig. 1:**
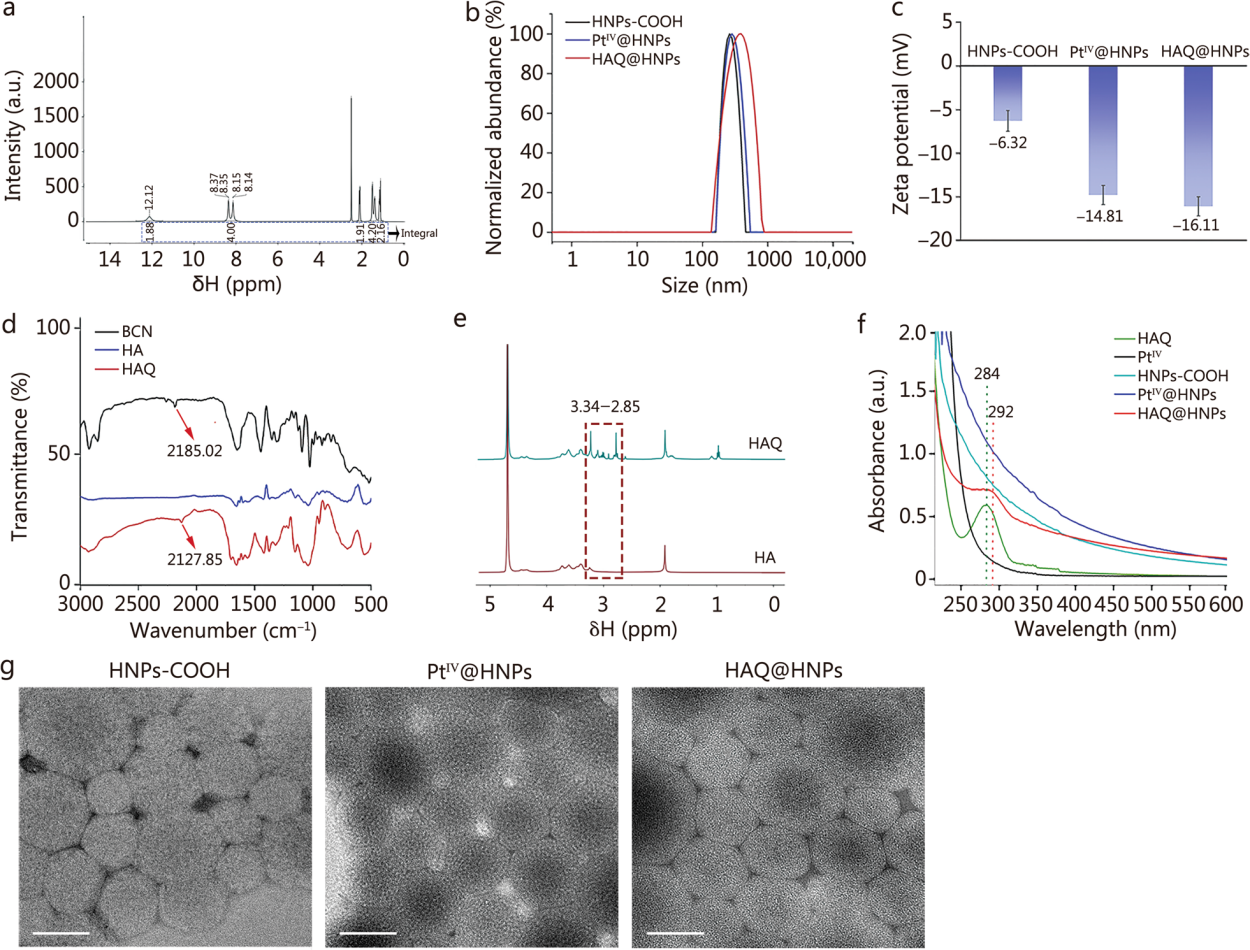
Characterization of pretargeted delivery system HAQ@HNPs. **a** ^1^H-NMR spectra of Pt^IV^. **b** DLS result of HNPs-COOH, Pt^IV^@HNPs, and HAQ@HNPs. **c** Zeta potential value of HNPs-COOH, Pt^IV^@HNPs, and HAQ@HNPs. **d** Infrared spectrum of BCN, HA, and HAQ. **e** ^1^H-NMR spectra of HA and HAQ.** f** UV–vis absorption spectra of HAQ, Pt^IV^, HNPs-COOH, Pt^IV^@HNPs, and HAQ@HNPs. **g** TEM images of HNPs-COOH, Pt^IV^@HNPs, and HAQ@HNPs. Scale bar = 200 nm. Data are expressed as mean ± SD. ^1^H-NMR ^1^H-nuclear magnetic resonance, DLS dynamic light scattering, UV–vis ultraviolet–visible, TEM transmission electron microscopy, SD standard deviation, HNPs-COOH carboxyl group-modified nanoparticles, Pt^IV^@HNPs Pt^IV^-loaded hydrogel nanoparticles, HAQ@HNPs Pt^IV^-loaded hydrogel nanoparticles with HAQ, BCN bicyclo[6.1.0]nonyne, HA hyaluronic acid, HAQ BCN-modified HA, Pt^IV^ platinum^IV^

Nano-hydrogel was selected as the main delivery platform to construct pretargeted delivery system. HAQ@HNPs were synthesized by photocrosslinking reaction. PEGDA (molecular weight 400–600 Da) and water formed nanoemulsions in oil with the help of surfactants, which were then irradiated with UV light in the presence of the photoinitiator 2-hydroxy-2-methylpropiophenone to obtain HNPs. Next, water-soluble oxaliplatin prodrug (Pt^IV^) was synthesized according to the reported method [33] and characterized by ^1^H-nuclear magnetic resonance (^1^H-NMR) and electrospray ionization mass spectrometry (ESI–MS). Amino-groups in Pt^IV^ were identified from a characteristic peak at δ8.37–δ8.14 ppm in ^1^H-NMR spectrum (Fig. 1a). ESI–MS spectrum showed a characteristic peak corresponding to [M-H]^−^ at *m/z* 532.9843 (Additional file 1: Fig. S1), jointly proving that Pt^IV^ was successfully synthesized. The HNPs (Pt^IV^@HNPs) were synthesized through the mixture between HNPs and Pt^IV^, which could reduce glutathione (GSH) level by valence state conversion from Pt^IV^ to Pt^II^ (oxaliplatin) [34–36]. Compared with HNPs-COOH, the hydrodynamic diameter of Pt^IV^@HNPs increased about 20 nm (Fig. 1b), and the zeta potential decreased up to –14.81 mV (Fig. 1c). HAQ@HNPs were prepared by modifying HAQ on the surface of Pt^IV^@HNPs through ester bridge. HAQ was synthesized by esterification between bicyclo[6.1.0]nonyne (BCN) and hyaluronic acid (HA) under catalysis of EDC. The successful coupling of HAQ was proved by Fourier transform infrared spectroscopy (FTIR) and ^1^H-NMR. Compared with the FTIR spectra of HA, the peak at 2127.85 cm^−1^ appeared (Fig. 1d), which was attributed to C≡C stretching vibration of BCN. As shown in Additional file 1: Fig. S2, the characteristic signals of HA were found in the ^1^H-NMR of HAQ at *δ*3.83 to *δ*3.34 ppm. Meanwhile, methylene hydrogens in HAQ were identified from a series of peaks from *δ*3.34 to *δ*2.85 ppm (^1^H-NMR spectrum), which belonged to the characteristic signals of BCN (Fig. 1e and Additional file 1: Fig. S2). Jointly, these results proved the successful synthesis of HAQ. Furthermore, the UV–vis spectrum showed that the fingerprint red-shift (8 nm) spectral peak observed at 292 nm for HAQ@HNPs was consistent with HAQ (284 nm), validating the successful preparation of HAQ@HNPs (Fig. 1f). TEM images presented a uniform spherical morphology of HAQ@HNPs with a size of approximately 300 nm in diameter (Fig. 1g), which was smaller than the result calculated from the dynamic light scattering (DLS) measurement with an average size of 350 nm, attributing to the adhesion effect of hydrogel. Moreover, the element mapping images exhibited an even distribution of carbon and platinum in HAQ@HNPs (Additional file 1: Fig. S3), which co-revealed the successful load of Pt^IV^. Compared with HNPs-COOH and Pt^IV^@HNPs, the surface of HAQ@HNPs became blurred and rough (Fig. 1g). And after the modification of HA, the zeta-potential decreased from −14.81 to −16.11 mV, indicating the successful modification of HA (Fig. 1c). The change of the particle size indicated that the nano-hydrogel was relatively stable during 7 d (Additional file 1: Fig. S4).

### Preparation and characterization of targeted delivery system (^223^Ra@HNPs)

#### Preparation and characterization of self-immolating molecule

**Fig. 2:**
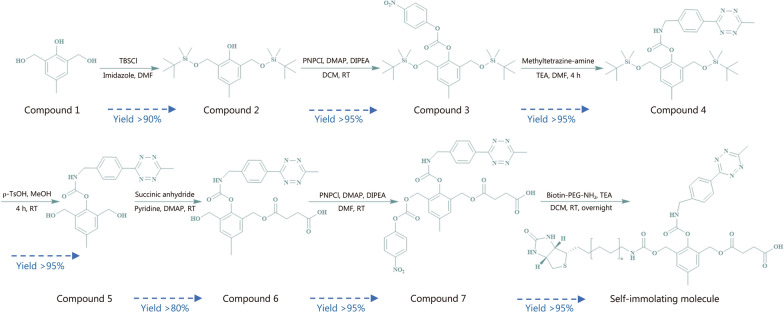
Synthesis of self-immolating molecule. Compound 1 is 2,6-bis(hydroxymethyl)-p-cresol. TBSCl tert-butyldimethylsilyl chloride, DMF N,N-dimethylformamide, PNPCl p-nitrophenyl chloroformate, DMAP 4-dimethylaminopyridine, DIPEA N,N-diisopropylethylamine, DCM dichloromethane, RT room temperature, TEA triethylamine, ρ-TsOH ρ-toluenesulfonic acid, MeOH methanol, PEG polyethylene glycol

Active targeted drug delivery system consists of 2 main parts: self-immolating molecule and nanocavity with radium. The cresol derivatives with substituents at ortho-benzylic positions could release 2 reactive termini simultaneously through a 1,4-electron elimination [34]. This tetrazine unit served as a handle to couple with strained dienophiles BCN through the highly efficient IEDDA reactions to induce self-immolating process. Once IEDDA reactions mediated by tetrazine occurred, a cyclisation step would occur to give the cresol derivatives with substituents at ortho-benzylic ends as the intermediate. Then, through a bidirectional 1,4-electron elimination, the self-immolating molecule would cleave the protective layer for Ra. As shown in Fig. 2, self-immolating molecule was synthesized via 7 chemical reaction steps, characterized by ^1^H/^13^C-NMR spectroscopy and ESI–MS as outlined in Additional file 1: Figs. S5-S11. Briefly, precursor compound 7 was synthesized in 6 steps from a 2,6-bis(hydroxymethyl)-p-cresol (compound 1) via transesterification reaction. As exhibited in Additional file 1: Fig. S10, the methyl peak in compound 7 was identified from a single peak at δ3.09 ppm (^1^H-NMR spectrum), which was assigned to the characteristic peaks of BCN. And double methylene peaks were found at δ2.89 and δ2.71 ppm (^1^H-NMR spectrum), assigned to the succinic acid group used to attach to hydrogel. High-resolution mass spectrometry (HRMS) showed a characteristic peak corresponding to [M-CO_2_ + H]^−^ at *m/z* 617.1994, jointly proving that compound 7 was successfully synthesized. Then, self-immolating molecule was synthesized by just one-step transesterification reaction under biotin-PEG-NH_2_ (average molecular weight 1000 Da). Compared with compound 7, extremely high PEG peaks attributed to biotin-PEG area were found at δ3.70 ppm (Additional file 1: Fig. S11), which demonstrated the successful preparation of self-immolating molecule. Notably, self-immolating molecule is a mixture with an average molecular weight of approximately 1400 Da, rather than a monomeric compound, which benefits a more stable nanostructure. Hence, the self-immolating molecule with the first lock [biotin receptor (BR) targeting] was constructed.

#### Preparation and characterizations of ^223^Ra@HNPs

**Fig. 3:**
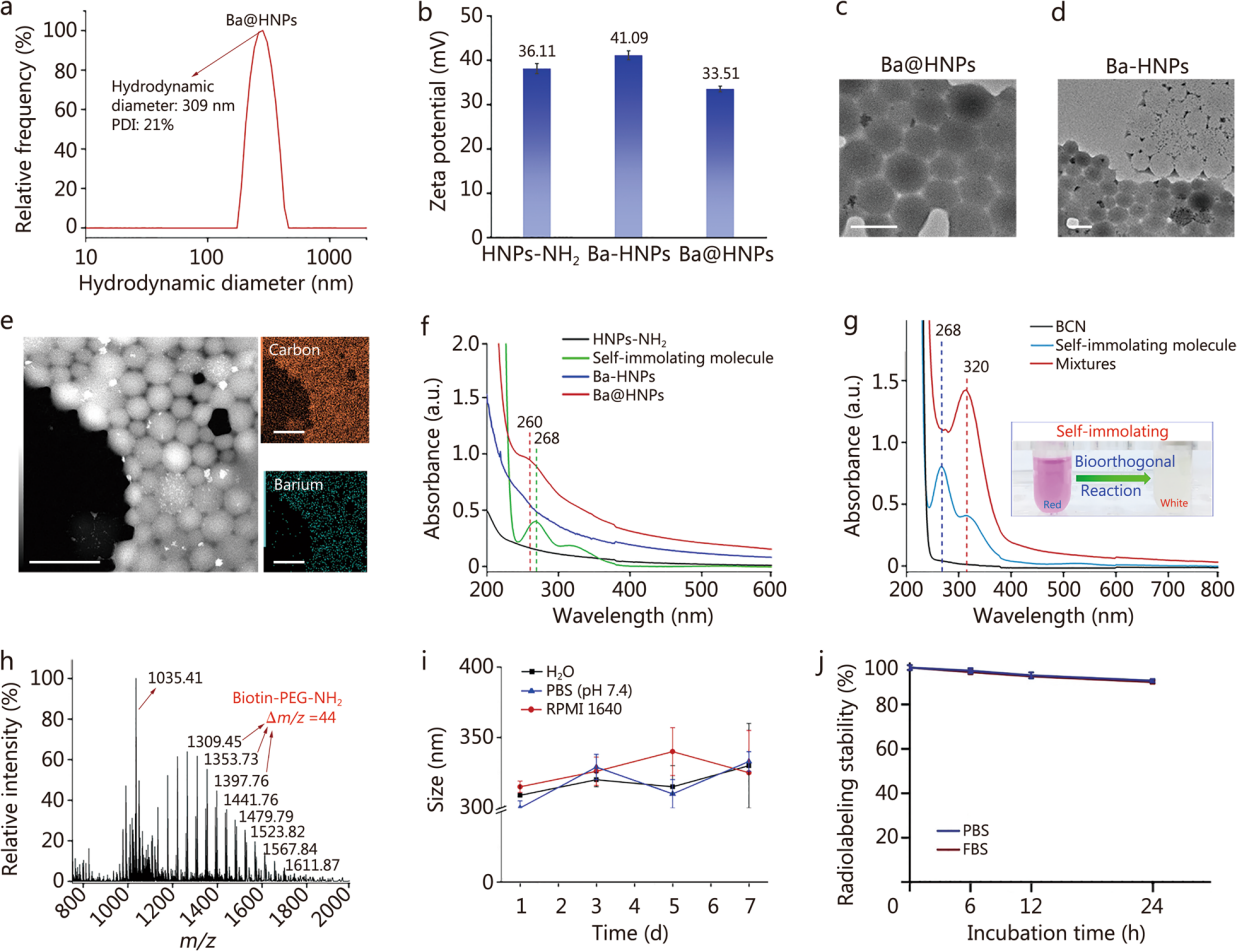
Characterization of targeted delivery system Ba@HNPs and ^223^Ra@HNPs. **a** DLS result of Ba@HNPs. **b** Zeta potential value of HNPs-NH_2_, Ba-HNPs, and Ba@HNPs.** c** TEM images of Ba@HNPs. Scale bar = 200 nm. **d** TEM images of Ba-HNPs. Scale bar = 500 nm. **e** TEM-elemental mapping of Ba@HNPs. Scale bar = 500 nm. **f** UV–vis absorption spectra of HNPs-NH_2_, self-immolating molecule, Ba-HNPs, and Ba@HNPs. **g** UV–vis absorption spectra of BCN, self-immolating molecule, and mixtures (self-immolating molecule and BCN). **h** ESI–MS (positive mode in methanol) of self-immolating molecule after IEEDA reaction.** i** DLS result of Ba@HNPs during 7 d in different media. **j** Radiolabeling stability of ^223^Ra@HNPs at 6, 12, and 24 h in different media (*n* = 3). Data are expressed as mean ± SD. DLS dynamic light scattering, TEM transmission electron microscopy, UV–vis ultraviolet–visible, BCN bicyclo[6.1.0]nonyne, ESI–MS electrospray ionization mass spectrometry, IEEDA inverse electron demand Diels–Alder, PDI polydispersity index, PBS phosphate-buffered saline, FBS fetal bovine serum, SD standard deviation, HNPs-NH_2_ amino-modified hydrogel nanoparticles, Ba-HNPs Ba^2+^-loaded hydrogel nanoparticles, Ba@HNPs Ba^2+^-loaded hydrogel nanoparticles with self-immolating molecule, PEG polyethylene glycol

Taking into account good biocompatibility and reproducible preparation, nano-hydrogel was selected as the delivery platform to construct targeted delivery system. A significant difference from pretargeted delivery system was the addition of 1-amino-3-butene hydrochloride, which allowed for coupling with self-immolating molecule via amide covalent bond. To minimize radiological hazards during physical and chemical characterization, Ba was used as a non-radioactive surrogate for Ra due to their similar chemical properties as group II alkaline earth metals. This substitution allows for safer handling while ensuring that the physicochemical properties observed with Ba-loaded nanohydrogel (Ba@HNPs) closely reflect those expected for the radium-loaded counterpart (^223^Ra@HNPs). Given that Ba^2+^ and Ra^2+^ share comparable ionic radii and coordination chemistry, their interactions with the nanohydrogel matrix should be analogous, making Ba a suitable model for pre-experimental validation. Compared with HNPs-NH_2_, the hydrodynamic diameter of Ba@HNPs increased about 15 nm (HNPs-NH_2_: 294 nm, Additional file 1: Fig. S12; Ba@HNPs: 309 nm, Fig. 3a), which resulted from the load of Ba and modification of self-immolating molecule. And after equipping with Ba^2+^, the zeta-potential increased from 36.11 to 41.09 mV, co-confirming the successful loading of Ba. Subsequently, the zeta-potential decreased over 7 mV, resulting from the block of amino group by amide bond on the surface of hydrogel (Fig. 3b). Intuitively, as exhibited in Fig. 3c, Ba@HNPs exhibited a uniform spherical morphology with a size of approximately 150–200 nm in diameter, which was still smaller than the result from the DLS measurement with an average size of 309 nm. Importantly, the transition state of preparation was captured by TEM, as shown in Fig. 3d, it was obvious to distinguish between the nanohydrogel loaded with Ba^2+^ and blank nanohydrogel. Notably, Ba element could not be washed away after coupling with self-immolating molecule on hydrogel, indicating the molecule could prevent the leakage of Ba. Moreover, the element mapping images also exhibited an even distribution of carbon and barium in Ba@HNPs (Fig. 3e). Furthermore, the ultraviolet–visible (UV–vis) spectrum showed that the fingerprint blue-shift (8 nm) spectral peak observed at 260 nm for Ba@HNPs was consistent with self-immolating molecule (268 nm), validating the successful modification with self-immolating molecule on HNPs (Fig. 3f). A summary table with particle sizes, zeta potentials, and loading content of above mentioned HNPs-COOH, Pt^IV^@HNPs, HAQ@HNPs, HNPs-NH_2_, Ba-HNPs, and Ba@HNPs was showed in Additional file 1: Table S1. In order to investigate the self-immolation process, BCN was mixed with self-immolating molecule. As shown in Fig. 3g, the characteristic absorption peak of self-immolating molecule at 268 nm disappeared after IEDDA reaction, and the color turned to be white from red within a few seconds, indicating the successful proceeding of IEDDA reaction. Besides, the Ba and Pt^IV^ release from nanogel were below 15% by 2 h, presenting a low release in PBS buffer. Remarkably, both drugs presented a controlled rapid release from approximately 15% to 60% upon mixture during 0.5–1.0 h, indicating encapsulation was removed by IEEDA reaction (Additional file 1: Fig. S13). As exhibited in the ESI–MS spectrum (Fig. 3h), the characteristic ion peaks (*m/z* = 1035.41) for IEDDA reaction products were marked by red arrow. And the normally distributed molecular ion peak with a mass-to-charge difference of 44 was observed, which belonged to the PEG fragment from post-immolation. UV–vis and ESI–MS spectrums co-proved that the efficient and successful proceeding of IEDDA reaction. The change of the particle size indicated that the Ba@HNPs was relatively stable during 7 d (Fig. 3i). Next, a similar method was used to load ^223^Ra, resulting in the targeted delivery system ^223^Ra@HNPs. The radiochemical yield (RCY) was determined to be (81.44 ± 1.36)% with over 95% radiochemical purity (RCP) by gamma counter. The radiolabeling stability assay demonstrated that ^223^Ra@HNPs maintained high stability in PBS and 10% FBS for up to 24 h, which was sufficient for killing tumor cells effectively (Fig. 3j).

### Cytotoxicity of HAQ/^223^Ra@HNPs in vitro

**Fig. 4:**
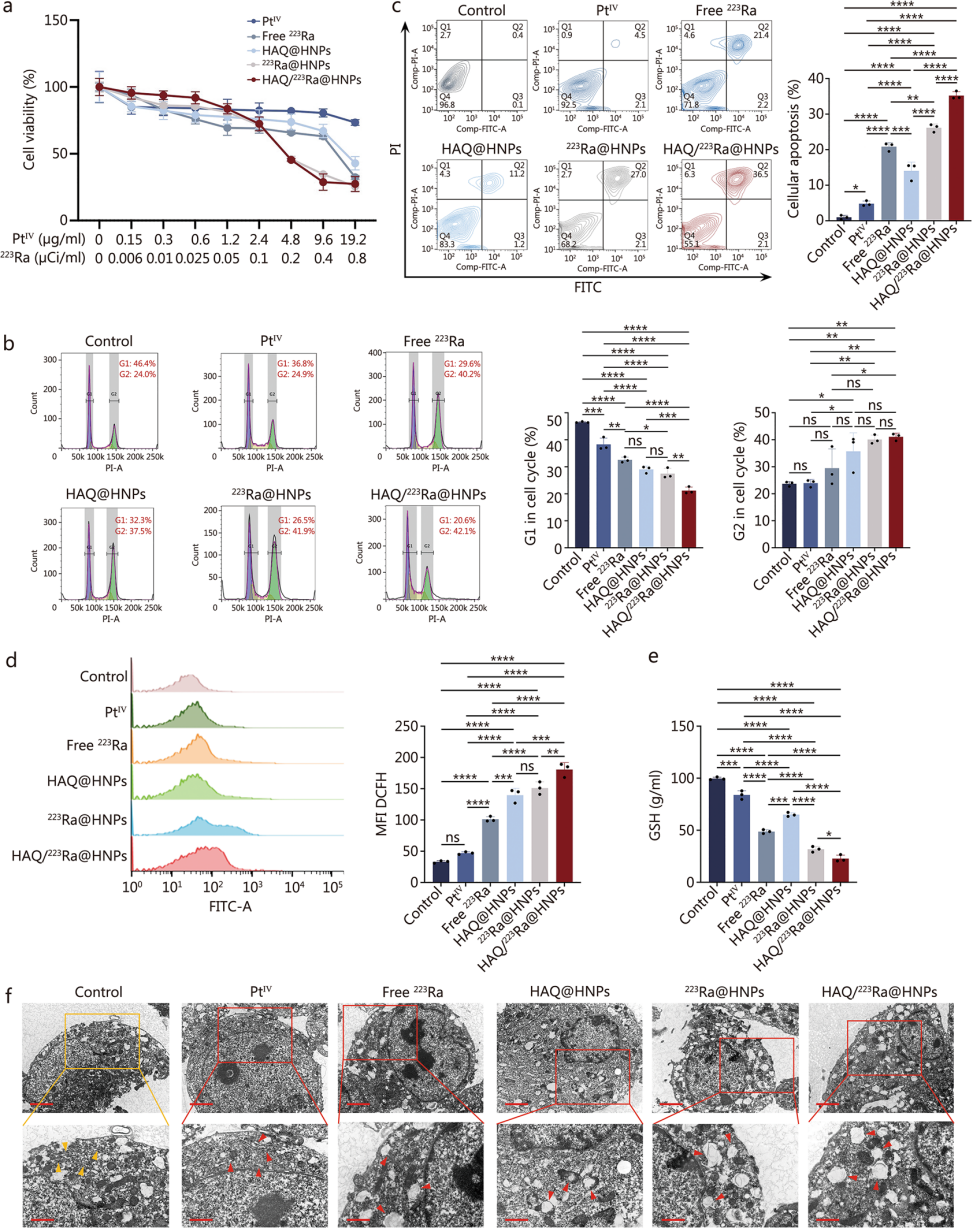
Cytotoxicity of HAQ/^223^Ra@HNPs in vitro. **a** Cell viability of B16/F10 cells under different treatments for 24 h (*n* = 4). **b** Cell cycle state distribution of B16/F10 cells after different treatments. **c** Apoptosis evaluation of B16/F10 cells under different treatments (*n* = 3). **d** FCM analysis of B16/F10 cells stained with DCFH-DA to reveal intracellular ROS generation after different treatments (*n* = 3). **e** GSH levels in B16/F10 cells after different treatments (*n* = 3). **f** Bio-TEM images of pristine B16/F10 cells (control) and the B16/F10 cells after different treatments for 24 h. Scale bar = 2 μm (upper)and 1 μm (lower). Yellow arrows mark reveal the normal ER, while red ones indicate the ER under stress state. Data are expressed as mean ± SD. ^*^P < 0.05, ^**^P < 0.01, ^***^P < 0.001, ^****^P < 0.0001, ns non-significant. ROS reactive oxygen species, FITC fluorescein isothiocyanate, PI propidium Iodide, GSH glutathione, SD standard deviation, Pt^IV^ platinum^IV^, HAQ@HNPs Pt^IV^-loaded hydrogel nanoparticles with HAQ, ^223^Ra@HNPs ^223^Ra-loaded hydrogel nanoparticles with self-immolating molecule, HAQ/^223^Ra@HNPs Pt^IV^-loaded hydrogel nanoparticles with HAQ and ^223^Ra-loaded hydrogel nanoparticles with self-immolating molecule, FCM flow cytometry, DCFH-DA 2’,7’-dichlorodihydrofluorescein diacetate, Bio-TEM Biological TEM

To evaluate the cytotoxic effects of HAQ/^223^Ra@HNPs, the pretargeted nanoplatform comprising both HAQ@HNPs (pretargeted nanohydrogel) and ^223^Ra@HNPs (radium-loaded targeted nanocapsules), on B16/F10 murine melanoma cells, a CCK-8 assay was performed to compare cell viability after different treatments with a gradient of doses for 24 h, including Pt^IV^, free ^223^Ra, HAQ@HNPs, ^223^Ra@HNPs, and HAQ/^223^Ra@HNPs. As shown in Fig. 4a, when the ^223^Ra loading was 0.2 µCi/ml and the Pt^IV^ content was 4.8 µg/ml, the survival rate of B16F10 cells after 24 h of HAQ/^223^Ra@HNPs treatment was approximately 50%, indicating antitumor cytotoxicity. Besides, additional in vitro experiments were conducted using the murine non-small cell lung cancer LLC cell line as well as HUVEC cell line to assess the potential cytotoxicity of HAQ/^223^Ra@HNPs. Consistent with the findings in B16/F10 cells (Fig. 4a), the nanoplatform demonstrated significant tumor cell killing in the LLC cells, while exhibiting relatively mild effects on HUVECs, suggesting a favorable therapeutic window (Additional file 1: Fig. S14). Based on the dose–response relationship, this concentration was selected for subsequent in vitro experiments. The cell cycle distribution was subsequently analyzed via FCM (Fig. 4b). In the control group, cells were distributed primarily in the G1 phase (46.4%). Compared to the control group, B16/F10 cells exposed to Pt^IV^, free ^223^Ra, HAQ@HNPs, and ^223^Ra@HNPs showed a reduction in G1 phase. Meanwhile, B16/F10 cells exposed to Free ^223^Ra, HAQ@HNPs, and ^223^Ra@HNPs showed an increase in G2 phase. Following HAQ/^223^Ra@HNPs treatment, a marked increase in the G2 phase (42.1%) and a significant reduction in the G1 population (20.6%) were observed, indicating that cell cycle arrest at the G2 checkpoint was induced, thereby inhibiting proliferation. To assess the induction of apoptosis, Annexin V/PI dual staining was conducted, followed by FCM analysis (Fig. 4c). A significantly greater proportion of apoptotic cells was observed in the HAQ/^223^Ra@HNPs group than in the other groups, confirming the apoptotic effects of the treatment.

To further elucidate the underlying mechanisms, intracellular reactive oxygen species (ROS) levels were measured using FCM with 2’,7’-dichlorodihydrofluorescein diacetate (DCFH-DA) staining. The results (Fig. 4d) revealed a substantial increase in ROS generation following treatment with HAQ/^223^Ra@HNPs, suggesting that oxidative stress plays a key role in tumoricidal activity. Since GSH is a crucial regulator of cellular redox homeostasis, intracellular GSH levels were also measured after different treatments. As shown in Fig. 4e, a significant depletion of GSH was observed following HAQ/^223^Ra@HNPs exposure, further supporting the hypothesis that ROS-mediated oxidative stress contributed to cytotoxicity and induced ER stress. To investigate ER stress, Biological TEM (Bio-TEM) was employed, as morphological changes in the ER serve as a hallmark of stress responses. As shown in Fig. 4f, red arrows indicate increased edema, expansion, vacuolization, and degranulation in the ER of the HAQ/^223^Ra@HNPs-treated group, compared to the typical ER morphology of B16/F10 cells in the control group. Therefore, HAQ/^223^Ra@HNPs exert potent cytotoxic effects on B16/F10 cells, underscoring their potential as a promising therapeutic agent for synergistic TAT and chemotherapy.

### Pretargeted study and antitumor evaluations of HAQ/^223^Ra@HNPs

**Fig. 5:**
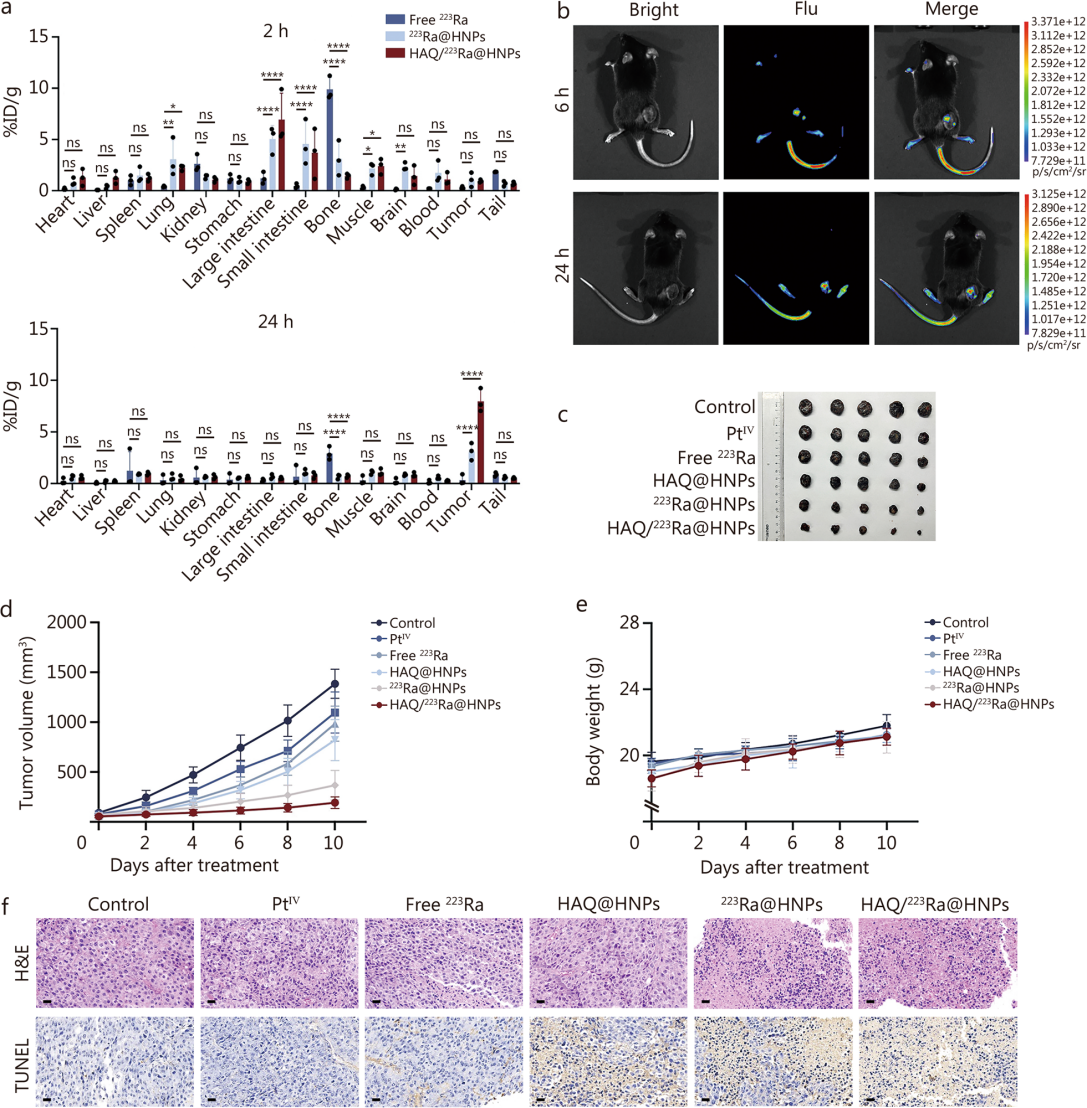
Pretargeted study and antitumor evaluation of HAQ/^223^Ra@HNPs. **a** Biodistribution of free ^223^Ra, ^223^Ra@HNPs, and HAQ/^223^Ra@HNPs in mice at 2 and 24 h injection (*n* = 3). **b** Representative fluorescence images of B16/F10 tumor-bearing mice post injection of HAQ/Cy7-^223^Ra@HNPs on 6 and 24 h. **c** Ex vivo tumor images from each group on day 10 in B16/F10 tumor-bearing mice (*n* = 5). **d** Tumor growth curves of B16/F10 tumor-bearing mice from different treatment groups (*n* = 5). **e** Body weights of B16/F10-bearing mice after different treatments (*n* = 5). **f** Representative H&E histopathological images and immunohistochemical analysis of TUNEL staining after different treatments. Scale bar = 20 μm. Data are expressed as mean ± SD. ^*^P < 0.05, ^**^P < 0.01, ^****^P < 0.0001, ns non-significant. Flu fluorescence, H&E hematoxylin and eosin, TUNEL terminal deoxynucleotidyl transferase dUTP nick-end labeling, SD standard deviation, Pt^IV^ platinum^IV^, HAQ@HNPs Pt^IV^-loaded hydrogel nanoparticles with HAQ, ^223^Ra@HNPs ^223^Ra-loaded hydrogel nanoparticles with self-immolating molecule, HAQ/^223^Ra@HNPs Pt^IV^-loaded hydrogel nanoparticles with HAQ and ^223^Ra-loaded hydrogel nanoparticles with self-immolating molecule

To evaluate the pretargeted potential and therapeutic efficacy of HAQ/^223^Ra@HNPs, a series of in vivo experiments were conducted using B16/F10 tumor-bearing mice. The biodistribution of radionuclides was first assessed by monitoring the radioactive accumulation in tumors and major organs at different time points (2, 6, 12, 24, 48, and 72 h post-injection) via γ-counter analysis (Fig. 5a; Additional file 1: Fig. S15). The results indicated that free ^223^Ra exhibited rapid clearance from the body, with a predominant accumulation in bone tissue within the initial few hours, consistent with its known osteotropic nature. In contrast, HAQ/^223^Ra@HNPs displayed higher tumor-to-organ uptake ratios, indicating improved selectivity and reduced systemic exposure. Similarly, the DAR and H&E images reveal a marked increase in tumor uptake of HAQ/^223^Ra@HNPs compared to free ^223^Ra at 72 h, also indicating enhanced tumor targeting capability (Additional file 1: Fig. S16). More importantly, the results also demonstrate that HAQ/^223^Ra@HNPs possess excellent tumor-targeting capability (Additional file 1: Fig. S17 and Table S2).

Additionally, a fluorescently-labeled analog, HAQ/Cy7-^223^Ra@HNPs, was intravenously administered to monitor its biodistribution (Fig. 5b). The enhanced tumor accumulation can be attributed to the precisely designed dual-lock mechanism, which ensures a two-step targeting: BR ligand-mediated targeting and covalent-mediated targeting. Upon reaching the tumor microenvironment, a bioorthogonal click chemistry reaction was triggered between the intratumorally injected HAQ@HNPs and the systemically delivered ^223^Ra@HNPs. This reaction induced the dissociation of the self-immolating molecule enabling the controlled release of ^223^Ra and Pt^IV^. The precisely localized release of these therapeutic agents enhanced the synergistic tumor-killing effects of TAT and chemotherapy, maximizing efficacy while minimizing systemic toxicity.

Consequently, the in vivo antitumor efficacy of HAQ/^223^Ra@HNPs was systematically assessed. B16/F10-bearing mice and LLC-bearing mice were randomly divided into 6 groups and subjected to different treatments, including control, Pt^IV^, free ^223^Ra, HAQ@HNPs, ^223^Ra@HNPs, and HAQ/^223^Ra@HNPs. The therapeutic responses were assessed over 10 d, after which the mice were euthanized, and tumor tissues were excised and weighed (Fig. 5c; Additional file 1: Fig. S18). Compared to the control group, a significant reduction in tumor weight was observed in the HAQ/^223^Ra@HNPs-treated group, demonstrating superior tumor growth suppression relative to other groups. Tumor growth curves were recorded for all groups (Fig. 5d). HAQ/^223^Ra@HNPs exhibited pronounced tumor growth suppression, correlating with its extended tumor residence time and sustained therapeutic effect, as also observed in LLC-bearing mice (Additional file 1: Fig. S19a, b). Both in B16/F10-bearing mice and LLC-bearing mice, no significant fluctuations in body weight were detected across all treatment groups, indicating that HAQ/^223^Ra@HNPs exerted minimal systemic toxicity (Fig. 5e and Additional file 1: Fig. S19c). Besides, H&E staining of major organs also revealed no visible damage to normal tissues (Additional file 1: Fig. S20). In terms of biosafety, liver and kidney function markers [alanine aminotransferase (ALT), aspartate aminotransferase (AST), blood urea nitrogen (BUN), and creatinine (CREA)], and complete blood count parameters [white blood cell (WBC), red blood cell (RBC), and platelet (PLT)] were also measured and showed negligible deviations, suggesting the non-toxic nature of HAQ/^223^Ra@HNPs (Additional file 1: Fig. S21).

To investigate the underlying mechanisms of tumor suppression, histopathological analyses, including H&E staining and terminal deoxynucleotidyl transferase dUTP nick-end labeling (TUNEL) assays, were performed on excised tumor tissues (Fig. 5f). H&E staining revealed that tumor tissues from the control and other groups retained relatively intact cellular architecture, whereas the HAQ/^223^Ra@HNPs-treated tumors exhibited extensive necrosis, characterized by nuclear condensation and cell shrinkage. TUNEL staining further confirmed that apoptotic activity was significantly elevated in the HAQ/^223^Ra@HNPs group, reinforcing the notion that tumor cell death was primarily induced through apoptosis and/or necrosis. Furthermore, immunofluorescence analysis on tumor tissues from each group was conducted to investigate relevant cell cycle regulatory pathways. The results showed a significant upregulation of p53 and its downstream effector p21 following HAQ/^223^Ra@HNPs treatment, indicating activation of the DNA damage response pathway (Additional file 1: Fig. S22). As a well-established regulatory axis of the cell cycle, the p53/p21 pathway plays a pivotal role in halting cell cycle progression, particularly by blocking the transition from G2 phase to mitosis, thus leading to G2/M phase arrest. This mechanism not only suppresses tumor cell proliferation but also facilitates the induction of programmed cell death when DNA damage is irreparable [37–39]. These findings provide further support for the contribution of G2/M arrest to the therapeutic efficacy of HAQ/^223^Ra@HNPs. Taken together, these findings demonstrated that HAQ/^223^Ra@HNPs not only exhibited excellent tumor-targeting ability and prolonged tumor retention but also effectively suppressed tumor growth via apoptosis-mediated cytotoxicity.

### ER stress response and immune activation induced by HAQ/^223^Ra@HNPs in vivo

**Fig. 6:**
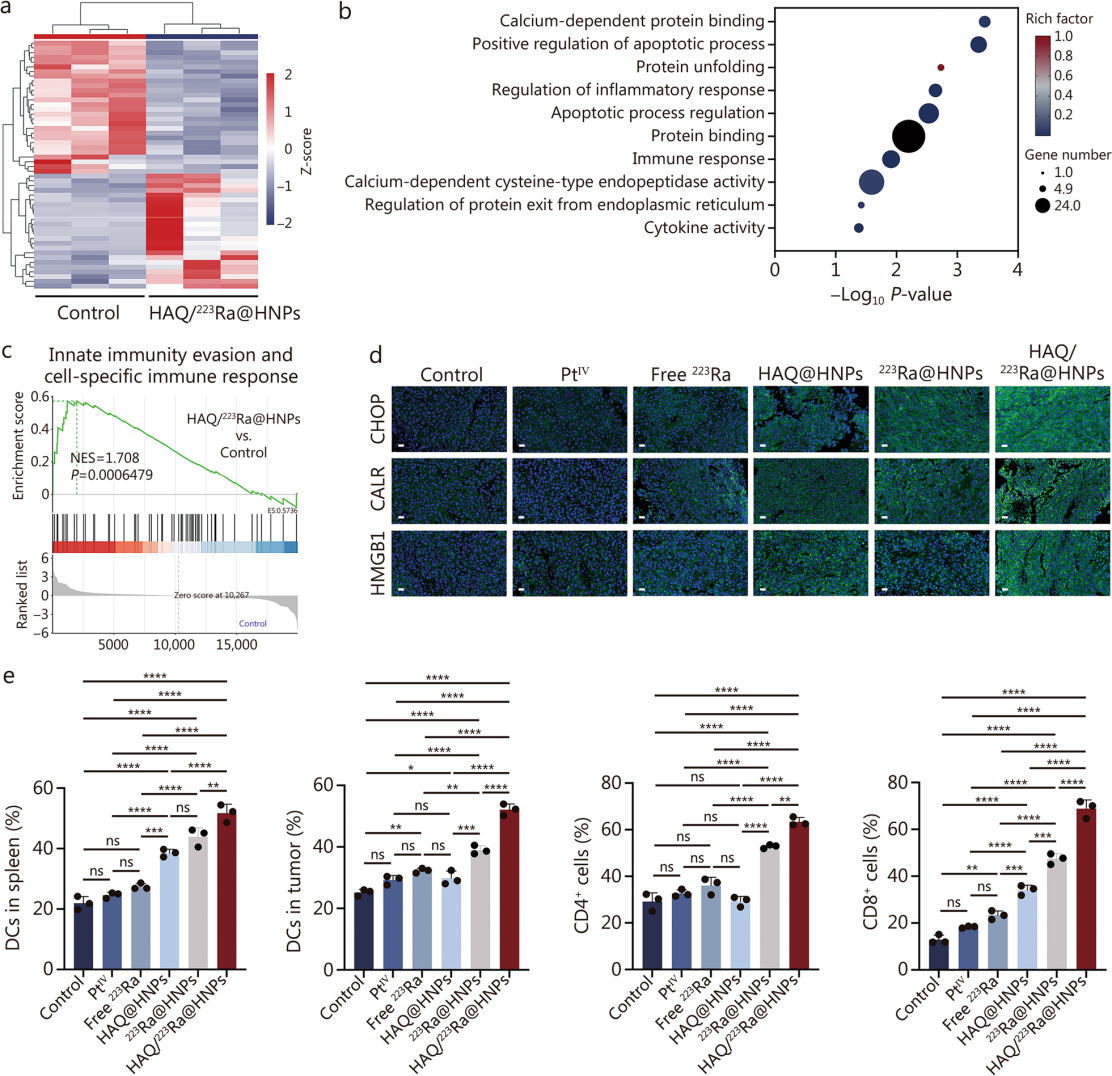
Endoplasmic reticulum stress response and immune responses induced by HAQ/^223^Ra@HNPs in vivo. **a** Heatmap of DEGs between the control and HAQ/^223^Ra@HNPs groups (*n* = 3). Red indicates higher expression levels with stronger positive correlation, while blue indicates lower expression levels with negative correlation. **b** GO enrichment analysis. **c** GSEA of the gene set “Innate immunity evasion and cell specific immune response”. **d** Representative immunofluorescence images of CHOP, CALR, and HMGB1 in tumor tissues from B16/F10 tumor-bearing mice after different treatments. **e** Statistical data of DCs maturation in the spleen, DCs maturation, CD4^+^ T cells, and CD8^+^ T cells in tumors from different groups (*n* = 3). Data are expressed as mean ± SD. ^*^P < 0.05, ^**^P < 0.01, ^***^P < 0.001, ^****^P < 0.0001, ns non-significant. SD standard deviation, DEGs differentially expressed genes, GO Gene Ontology, GSEA gene set enrichment analysis, CHOP C/EBP homologous protein, CALR calreticulin, HMGB1 high mobility group box 1, DC dendritic cell, Pt^IV^ platinum^IV^, HAQ@HNPs Pt^IV^-loaded hydrogel nanoparticles with HAQ, ^223^Ra@HNPs ^223^Ra-loaded hydrogel nanoparticles with self-immolating molecule, HAQ/^223^Ra@HNPs Pt^IV^-loaded hydrogel nanoparticles with HAQ and ^223^Ra-loaded hydrogel nanoparticles with self-immolating molecule

Having investigated the molecular mechanisms underlying the antitumor effects of HAQ/^223^Ra@HNPs, mice were sacrificed after 7 d of treatment, and RNA sequencing (RNA-seq) analysis and transcriptomic profiling were performed on the tumor tissues following treatment. Differentially expressed genes (DEGs) between the control and HAQ/^223^Ra@HNPs-treated groups were visualized using hierarchical clustering (Fig. 6a). A distinct gene expression pattern was observed, suggesting that HAQ/^223^Ra@HNPs induced profound transcriptional changes. Gene Ontology (GO) enrichment analysis was further performed to elucidate the biological pathways affected by HAQ/^223^Ra@HNPs treatment (Fig. 6b). The results revealed significant enrichment in pathways related to calcium-dependent protein binding, apoptotic process regulation, regulation of inflammatory response, and immune response, indicating that HAQ/^223^Ra@HNPs exerted therapeutic effects via the induction of cellular stress and immune modulation. Gene set enrichment analysis (GSEA) was applied to identify immune-related pathways influenced by HAQ/^223^Ra@HNPs treatment. Notably, a strong enrichment in the “innate immunity evasion and cell-specific immune response” gene set was detected in the HAQ/^223^Ra@HNPs-treated group compared with the control group (Fig. 6c), further confirming the role of this nanoplatform in enhancing antitumor immunity.

Given the established role of ER stress in ICD [40, 41], key ER stress markers were analyzed via immunofluorescence staining of tumor tissues. The results revealed significantly elevated expression levels of ER stress marker C/EBP homologous protein (CHOP) (Fig. 6d), glucose-regulated protein 78 (GRP78), and PKR-like endoplasmic reticulum kinase (PERK) (Additional file 1: Fig. S23) in tumor tissues from the HAQ/^223^Ra@HNPs group, suggesting that alpha radiation effectively induces ER stress and activates the downstream unfolded protein response (UPR) pathway. Subsequently, as a result of ER stress, the translocation of calreticulin (CALR) to the cell surface as the “eat-me” signal was directly detected in differently treated groups [42]. Significant green fluorescent signals were observed in the tumor tissues, indicating that HAQ/^223^Ra@HNPs successfully induced CALR exposure in the cell plasma membrane (Fig. 6d). A significant upregulation of high mobility group box 1 (HMGB1) was also detected in the HAQ/^223^Ra@HNPs-treated group, indicating the induction of ER stress and ICD, which contributed to the activation of antitumor immunity (Fig. 6d). Moreover, we also observed a marked increase in PD-L1 expression in the HAQ/^223^Ra@HNPs group (Additional file 1: Fig. S24), as confirmed by immunofluorescence analysis.

To evaluate dendritic cells (DCs) maturation and tumor-infiltrating lymphocytes, FCM was conducted (Fig. 6e; Additional file 1: Fig. S25). The proportion of mature DCs was significantly increased in both the spleen and tumor following HAQ/^223^Ra@HNPs treatment, indicating enhanced antigen presentation capacity. A substantial elevation in CD4⁺ and CD8⁺ T cells within the HAQ/^223^Ra@HNPs-treated tumors was also observed. Besides, blood samples were collected to detect the secretion of cytokines tumor necrosis factor-α (TNF-α) and interferon-γ (IFN-γ) (Additional file 1: Fig. S26), which were markedly elevated in the HAQ/^223^Ra@HNPs group. These results suggested that HAQ/^223^Ra@HNPs not only triggered robust ER stress responses but also facilitated adaptive immune activation, further amplifying antitumor efficacy.

### Antitumor evaluations of combination treatment with HAQ/^223^Ra@HNPs and anti-PD-L1 on metastatic tumor model

**Fig. 7:**
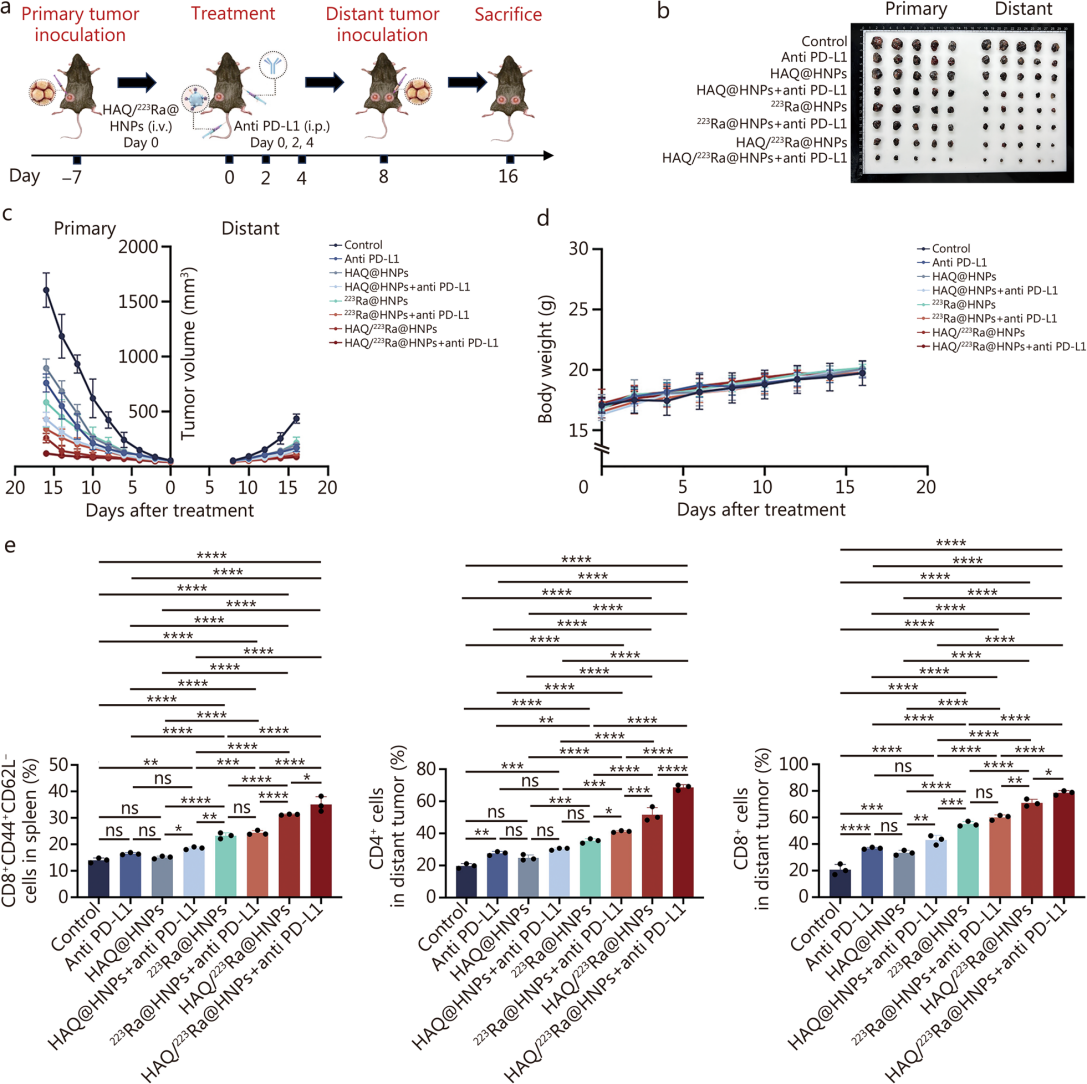
Antitumor evaluations of combination treatment with HAQ/^223^Ra@HNPs and anti PD-L1 on metastasis tumor models.** a** Treatment schedule for in vivo combination treatment. **b** Photos of excised primary and distant tumors from the mice after 16 d in corresponding treatment groups (*n* = 5). **c** Individual tumor growth curves of mice from different treatment groups (*n* = 5). **d** Body weight of mice after different treatments (*n* = 5). **e** Statistical data of T_EM_ cells in spleen, CD4^+^ T cells and CD8^+^ T cells in distant tumors. Data are expressed as mean ± SD. ^*^P < 0.05, ^**^P < 0.01, ^***^P < 0.001, ^****^P < 0.0001, ns non-significant. SD standard deviation, T_EM_ Immune effector memory T cells, Pt^IV^ platinum^IV^, HAQ@HNPs Pt^IV^-loaded hydrogel nanoparticles with HAQ, ^223^Ra@HNPs ^223^Ra-loaded hydrogel nanoparticles with self-immolating molecule, HAQ/^223^Ra@HNPs Pt^IV^-loaded hydrogel nanoparticles with HAQ and.^223^Ra-loaded hydrogel nanoparticles with self-immolating molecule, PD-L1 programmed death-ligand 1

The findings above demonstrated that HAQ/^223^Ra@HNPs effectively enhanced tumor immunogenicity. To further assess its potential in stimulating systemic antitumor immunity and its efficacy in combination with immune checkpoint blockade (ICB) therapy for controlling metastatic progression, a distant metastatic tumor model was established (Fig. 7a). After administration of different treatments, tumor excision and weight measurements were performed on day 16 (Fig. 7b; Additional file 1: Fig. S27). The results demonstrated that the “HAQ/^223^Ra@HNPs + anti-PD-L1” group significantly suppressed both primary and distant tumor growth, achieving superior therapeutic efficacy compared to monotherapies. This finding highlighted the synergistic effect of the combination regimen in controlling metastatic tumor progression. Tumor growth kinetics were recorded for each treatment group (Fig. 7c). A sustained suppression of tumor growth was observed in the “HAQ/^223^Ra@HNPs + anti-PD-L1” group, while mice treated with single-agent therapy exhibited a moderate tumor inhibition effect. Furthermore, no significant changes in body weight were detected (Fig. 7d), indicating that the combination therapy was well-tolerated and did not induce systemic toxicity.

To assess whether systemic antitumor immunity was activated by the treatment, immune cell populations were analyzed using FCM. A significant increase in effector memory T cells (T_EM_, CD8^+^CD44^+^CD62L^−^) was detected in the spleen following combination therapy (Fig. 7e; Additional file 1: Fig. S28a), suggesting the induction of long-term immune memory. Additionally, CD4⁺ T cell infiltration was markedly enhanced in the metastatic tumor microenvironment (Fig. 7e; Additional file 1: Fig. S28b), and a dramatic increase in CD8⁺ T cells was observed in the “HAQ/^223^Ra@HNPs + anti-PD-L1” group (Fig. 7e; Additional file 1: Fig. S28c), indicating a robust cytotoxic T cell response. These findings collectively demonstrated that HAQ/^223^Ra@HNPs, when combined with anti-PD-L1 therapy, effectively inhibited both primary and metastatic tumor growth by enhancing systemic immune responses and promoting tumor-specific T cell activation. This strategy holds great potential for metastatic cancer treatment, offering an effective approach for overcoming tumor immune evasion and enhancing therapeutic outcomes.

## Discussion

**Fig. 8:**
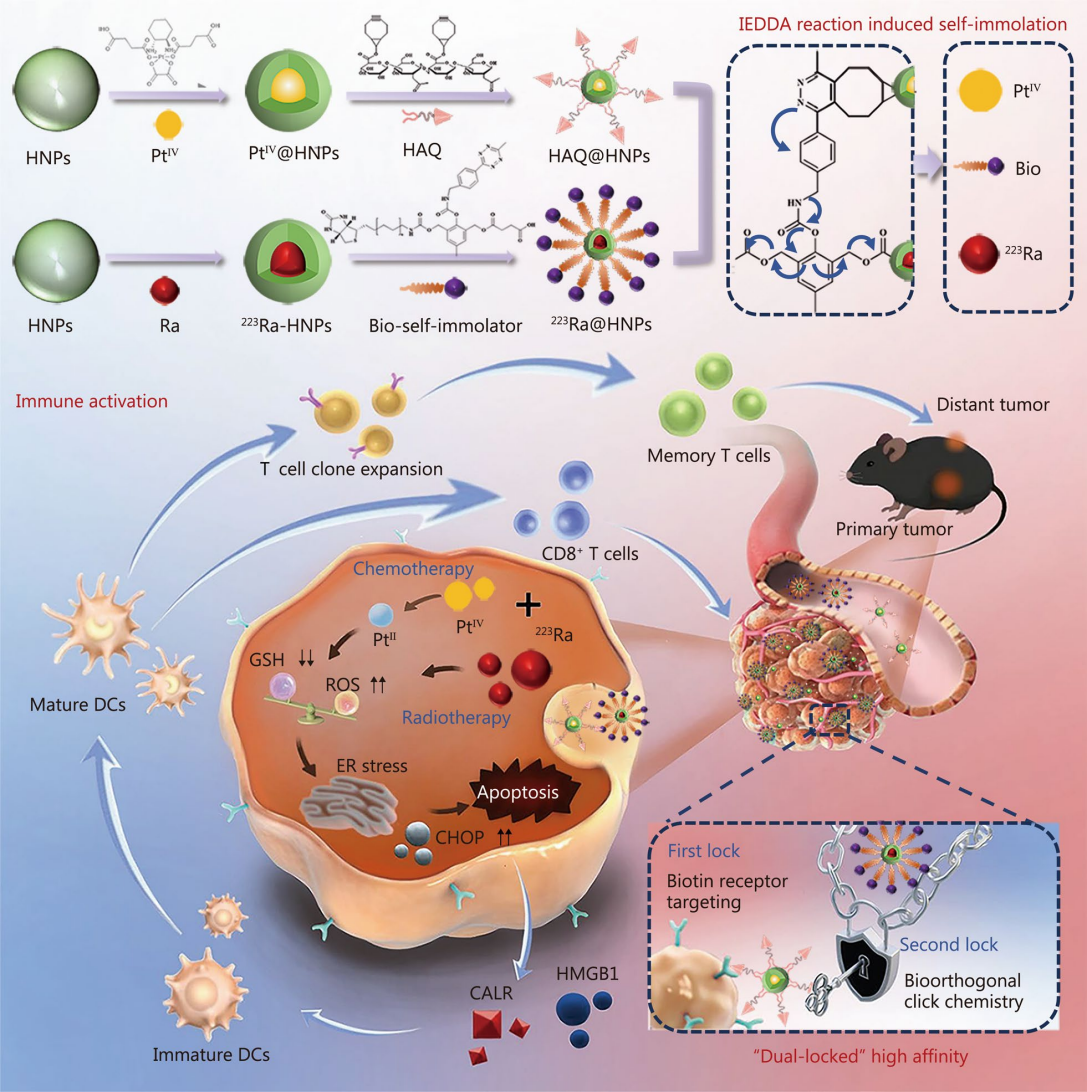
Schematic illustration of synthesis and therapeutic mechanism of dual-locked targeted alpha-emitter. HNPs hydrogel nanoparticles, Pt platinum, Bio biotin, IEDDA inverse electron demand Diels–Alder, DC dendritic cell, CALR calreticulin, HMGB1 high mobility group box 1, ROS reactive oxygen species, ER endoplasmic reticulum, GSH glutathione, CHOP C/EBP homologous protein

The strategy employed Pt^IV^-loaded HNPs as the primary platform of the pretargeting delivery system (HAQ@HNPs), and ^223^Ra-loaded nanocavities protected by self-inert spacers (self-immolating molecules) served as a targeted delivery system (^223^Ra@HNPs). As shown in Fig. 8, after encountering at tumor sites under biotin and HA guidance between the pretargeting and targeted delivery systems, the dissociation is rapidly triggered by the IEDDA reaction, releasing ^223^Ra and Pt^IV^, thus achieving the purpose of targeted alpha therapy with chemotherapy to kill the tumor directly.

In our experiments, beyond the direct cytotoxicity, both delivery systems loaded with ^223^Ra and Pt^IV^ promote the consumption of GSH in the cell, breaking the redox homeostasis, amplifying the ER stress. Especially, immunofluorescence analysis demonstrated that HAQ/^223^Ra@HNPs treatment markedly increased the expression of ER stress-related proteins (CHOP, GRP78, and PERK) and ICD markers (CALR and HMGB1), accompanied by a significant upregulation of PD-L1 in tumor tissues. It is well-known that radiation-induced stress leads to PD-L1 upregulation, resulting in potential immune escape [43]. Besides, GRP78, as a canonical marker of ER stress [44], has also been reported to play an upstream role in the initiation of ICD [45] and the upregulation of PD-L1 [46]. PERK, one of the 3 major branches of the UPR, is a critical sensor that can simultaneously promote apoptotic signaling through the activating transcription factor 4 (ATF4)/CHOP axis [47] and also upregulate PD-L1 expression [46]. Taken together, these findings suggest that HAQ/^223^Ra@HNPs-induced ER stress exerts a dual effect in antitumor immunity: it promotes ICD and enhances tumor immunogenicity, while also contributing to PD-L1 upregulation. We suppose that combining TAT with ICB may therefore yield durable immune activation and achieve long-term tumor suppression. Fortunately, consistent with this hypothesis, when further combined with PD-L1 antibody, we found that TAT can produce long-term and sustained immune effects to effectively inhibit tumor growth, metastasis or recurrence. These results form the conceptual foundation of our proposed, alpha radiation immunotherapy (ARIT), a therapeutic paradigm in which high-LET α-particle irradiation not only kills tumor cells directly but also reprograms the tumor microenvironment through immunogenic stress signaling.

In addition to mechanistic insights, it is worth emphasizing that accurate dosimetry remains a critical aspect of TAT. The DAR images of our result clearly demonstrate heterogeneous intratumoral distribution of radioactivity, which reflects one of the key challenges in ^223^Ra-based dosimetry: spatial dose heterogeneity at the microscopic level. Herein, based on the biodistribution data, the TIA and absorbed dose were calculated to estimate quantitative dose of HAQ/^223^Ra@HNPs. Due to the lack of mouse-specific S-values in existing dosimetry software (MIRDcalc: https://mirdsoft.org/mirdcalc) and Open-Access databases (OpenDose: https://opendose.polsl.pl/), which are largely based on anthropomorphic human phantoms, a simplified dosimetric approach was adopted. Specifically, we assumed all tissues to be equivalent to soft tissue and utilized MIRDcalc to estimate absorbed dose by applying standard soft-tissue S-values. Undeniably, the dosimetry method based on MIRD is a rough estimate and does not fully capture the detailed radiation dose distribution, especially at the microscopic level. Monte Carlo simulations, which can track the stochastic trajectories and interactions of individual particles within complex anatomical geometries [48–51], should be further elaborated on TAT dosimetry.

Collectively, this dual-locked targeted alpha-particle nanoplatform, integrating ARIT and chemotherapy, has exhibited exceptional therapeutic efficacy. Leveraging synergistic mechanisms, it not only enhances tumor eradication but also offers a promising strategy to advance the clinical translation of alpha-particle-based therapies.

## Conclusions

In this study, a dual-locked pretargeted nanoplatform was successfully developed to overcome the challenges associated with TAT, particularly the radionuclide leakage and nonspecific toxicity of ^223^Ra. By leveraging the bioorthogonally activated IEDDA reaction, the system enabled precise tumor targeting and controlled release of ^223^Ra and Pt^IV^, achieving enhanced therapeutic efficacy with minimal off-target effects. The combination of ARIT and chemotherapy not only directly eliminated tumor cells but also disrupted redox homeostasis, induced ER stress, and promoted ICD, leading to a robust activation of the antitumor immune response and further synergizing with immunotherapy. The findings provide strong evidence for the potential of dual-locked pretargeted strategies to enhance the clinical translation of TAT by addressing key limitations, including radionuclide retention, bioavailability, and systemic antitumor effects. This work not only expands the therapeutic landscape of TAT but also lays the groundwork for the development of next-generation precision-targeted radiopharmaceuticals.

## Supplementary Information

**Additional file 1**. **Methods. Fig. S1** ESI-MS spectrum of Pt^IV^. **Fig. S2** ^1^H-NMR spectrum of HA (**a**) and HAQ (**b**) in D_2_O. **Fig. S3** TEM-elemental mapping of HAQ@HNPs. **Fig. S4** The stability of the HAQ@HNPs incubated in different media. **Fig. S5** Characterization of compound 2. **Fig. S6** Characterization of compound 3. **Fig. S7** Characterization of compound 4. **Fig. S8** Characterization of compound 5. **Fig. S9** Characterization of compound 6. **Fig. S10** Characterization of compound 7. **Fig. S11** ^1^H-NMR spectrum of self-immolating spacer in methanol-d*4*. **Fig. S12** DLS result of HNPs-NH_2_ and Ba-HNPs. **Fig. S13** The release of Ba^2+^ and Pt^IV^ from nanogel. **Fig. S14** Cytotoxicity of HAQ/^223^Ra@HNPs in vitro. **Fig. S15** Biodistribution profile. **Fig. S16** Representative DAR and H&E images of frozen tissue sections from B16/F10-bearing mice following different treatments at 72 h post-injection. **Fig. S17** Time-activity curves and fitted functions for biodistribution of ^223^Ra and HAQ/^223^Ra@HNPs. **Fig. S18** Ex vivo tumor weights from each group at day 10 (*n* = 5) in B16/F10 tumor-bearing mice. **Fig. S19** Antitumor evaluations of HAQ/^223^Ra@HNPs in LLC tumor-bearing mice. **Fig. S20** Representative histological images of the main organs in B16/F10-bearing mice after different treatments, stained with hematoxylin and eosin. **Fig. S21** Hematological analysis was performed on blood withdrawn from B16/F10 tumor-bearing mice in corresponding treatment groups at the terminal of study (*n* = 5). **Fig. S22** Immunofluorescence staining of p53 and p21 in tumor tissues following different treatments. **Fig. S23** Immunofluorescence staining of GRP78 and PERK in tumor tissues following different treatments. **Fig. S24** Immunofluorescence staining of PD-L1 in tumor tissues following different treatments. **Fig. S25** Representative flow cytometry plots showing DCs maturation in the spleen, DCs maturation, CD4^+^ T cells, and CD8^+^ T cells in tumors from different groups (*n* = 3). **Fig. S26** The secretion of cytokines TNF-α and IFN-γ. **Fig. S27** Ex vivo tumor weight of excised primary and distant tumors from the mice after 16 d in corresponding groups (*n* = 5). **Fig. S28** Representative flow cytometry plots showing T_EM_ cells in spleen (**a**), CD4^+^ T cells (**b**), and CD8^+^ T cells (**c**) in distant tumors from different groups (*n* = 3). **Table S1** Characteristics of the nanoparticles (mean ± SD). **Table S2** Absorbed doses in each organ after administration of ^223^Ra and HAQ/^223^Ra@HNPs (Gy).

## Data Availability

The data that support the findings of this study are available from the corresponding author upon reasonable request.

## Abbreviations

ALT: Alanine aminotransferase
ARIT: Alpha radiation immunotherapy
AST: Aspartate aminotransferase
ATF4: Activating transcription factor 4
α-ZrP: Alpha-zirconium phosphate
Ba: Barium
BCN: Bicyclo[6.1.0]nonyne
Bio-TEM: Biological TEM
BR: Biotin receptor
BUN: Blood urea nitrogen
CALR: Calreticulin
CCK-8: Cell counting kit-8
CHOP: C/EBP homologous protein
CREA: Creatinine
DAR: Digital autoradiography
DC: Dendritic cell
DCM: Dichloromethane
DCFH-DA: 2’,7’-Dichlorodihydrofluorescein diacetate
DEGs: Differentially expressed genes
DIPEA: N,N-diisopropylethylamine
DLS: Dynamic light scattering
DMAP: 4-Dimethylaminopyridine
DMF: N,N-dimethylformamide
DMSO: Dimethyl sulfoxide
D_2_O: Deuterium oxide
EDC: 1-Ethyl-3-(3-dimethylaminopropyl) carbodiimide
EPR: Enhanced permeability and retention
ER: Endoplasmic reticulum
ESI–MS: Electrospray ionization mass spectrometry
FBS: Fetal bovine serum
FITC: Fluorescein isothiocyanate
FTIR: Fourier transform infrared spectroscopy
FCM: Flow cytometry
GRP78: Glucose-regulated protein 78
GSEA: Gene set enrichment analysis
GSH: Glutathione
GO: Gene Ontology
HA: Hyaluronic acid
H&E: Hematoxylin and eosin
HMGB1: High mobility group box 1
HNPs: Hydrogel nanoparticles
HRMS: High-resolution mass spectrometry
HUVEC: Human umbilical vein endothelial cell
H₂O₂: Hydrogen peroxide
ICB: Immune checkpoint blockade
ICD: Immunogenic cell death
IEDDA: Inverse electron demand Diels–Alder
IFN-γ: Interferon-γ
LDH: Layered double hydroxide
LET: Linear energy transfer
LLC: Lewis lung carcinoma
MeOH: Methanol
mCRPC: Metastatic castration-resistant prostate cancer
MIRD: Medical internal radiation dose
NHS: N-Hydroxysuccinimide
^1^H-NMR: ^1^H-nuclear magnetic resonance
PBS: Phosphate-buffered saline
PDI: Polydispersity index
PD-L1: Programmed death-ligand 1
PEG: Polyethylene glycol
PEGDA: Poly (ethylene glycol) diacrylate
PERK: PKR-like endoplasmic reticulum kinase
PNPCl: P-nitrophenyl chloroformate
PLT: Platelet
PI: Propidium iodide
PNPCl: P-nitrophenyl chloroformate
Pt: Platinum
Pt^IV^: Platinum^IV^
^223^Ra: Radium-223
^223^Ra@HNPs: ^223^Ra-loaded hydrogel nanoparticles
RBC: Red blood cell
RBE: Relative biological effectiveness
RCP: Radiochemical purity
RCY: Radiochemical yield
RNA-seq: RNA sequencing
ROS: Reactive oxygen species
RT: Room temperature
SPION: Superparamagnetic iron-oxide
TAT: Targeted alpha therapy
TBSCl: Tert-Butyldimethylsilyl chloride
TEA: Triethylamine
T_EM_: Immune effector memory T cells
TEM: Transmission electron microscopy
TIA: Time-integrated activity
TiO_2_: Titanium dioxide
TNF-α: Tumor necrosis factor-α
TUNEL: Terminal deoxynucleotidyl transferase dUTP nick-end labeling
UPR: Unfolded protein response
UV–vis: Ultraviolet–visible
WBC: White blood cell

## References

[1] Parker C, Lewington V, Shore N, Kratochwil C, Levy M, Lindén O, et al. Targeted alpha therapy, an emerging class of cancer agents: a review. JAMA Oncol. 2018;4(12):1765–72. 30326033 10.1001/jamaoncol.2018.4044

[2] Sgouros G. Alpha-particles for targeted therapy. Adv Drug Deliv Rev. 2008;60(12):1402–6. 18541332 10.1016/j.addr.2008.04.007

[3] Hall EJ, Hei TK. Genomic instability and bystander effects induced by high-LET radiation. Oncogene. 2003;22(45):7034–42. 14557808 10.1038/sj.onc.1206900

[4] Sgouros G. α-particle-emitter radiopharmaceutical therapy: resistance is futile. Cancer Res. 2019;79(21):5479–81. 31676677 10.1158/0008-5472.CAN-19-2806

[5] Parker C, Nilsson S, Heinrich D, Helle SI, O’Sullivan JM, Fosså SD, et al. Alpha emitter radium-223 and survival in metastatic prostate cancer. N Engl J Med. 2013;369(3):213–23. 23863050 10.1056/NEJMoa1213755

[6] Dizdarevic S, Mccready R, Vinjamuri S. Radium-223 dichloride in prostate cancer: proof of principle for the use of targeted alpha treatment in clinical practice. Eur J Nucl Med Mol Imaging. 2020;47(1):192–217. 31471713 10.1007/s00259-019-04475-5

[7] Price EW, Orvig C. Matching chelators to radiometals for radiopharmaceuticals. Chem Soc Rev. 2014;43(1):260–90. 24173525 10.1039/c3cs60304k

[8] Lankoff A, Czerwińska M, Walczak R, Karczmarczyk U, Tomczyk K, Brzóska K, et al. Design and evaluation of ^223^Ra-labeled and anti-PSMA targeted NaA nanozeolites for prostate cancer therapy-part II. Toxicity, pharmacokinetics and biodistribution. Int J Mol Sci. 2021;22(11):5702. 34071854 10.3390/ijms22115702 PMC8198605

[9] Czerwińska M, Fracasso G, Pruszyński M, Bilewicz A, Kruszewski M, Majkowska-Pilip A, et al. Design and evaluation of ^223^Ra-labeled and anti-PSMA targeted NaA nanozeolites for prostate cancer therapy-part I. Materials. 2020;13(17):3875. 32887308 10.3390/ma13173875 PMC7504699

[10] Wang Y, Zhou Y, Zhai D, Deng H, Chen X. Design and in vitro evaluation of ^223^Ra/^99^mTc-loaded spherical nano-hydroxyapatite in bone tumor therapy. Nanomedicine. 2024;19(17):1557–67. 39011932 10.1080/17435889.2024.2365127 PMC11321400

[11] Suchánková P, Kukleva E, Štamberg K, Nykl P, Sakmár M, Vlk M, et al. Determination, modeling and evaluation of kinetics of ^223^Ra sorption on hydroxyapatite and titanium dioxide nanoparticles. Materials. 2020;13(8):1915. 32325792 10.3390/ma13081915 PMC7216037

[12] Gemini-Piperni S, Ricci-Junior E, İlem-Özdemir D, Da Silva Batista B, Alencar LMR, Rossi AM, et al. Nano-hydroxyapatite radiolabeled with radium dichloride [^223^Ra] RaCl_2_ for bone cancer targeted alpha therapy: in vitro assay and radiation effect on the nanostructure. Colloids Surf B Biointerfaces. 2023;223:113174. 36746067 10.1016/j.colsurfb.2023.113174

[13] Suchánková P, Kukleva E, Nykl E, Nykl P, Sakmár M, Vlk M, et al. Hydroxyapatite and titanium dioxide nanoparticles: radiolabelling and in vitro stability of prospective theranostic nanocarriers for ^223^Ra and ^99^mTc. Nanomaterials. 2020;10(9):1632. 32825280 10.3390/nano10091632 PMC7558198

[14] Ondrák L, Ondrák Fialová K, Sakmár M, Vlk M, Štamberg K, Drtinová B, et al. Preparation and characterization of alpha-zirconium phosphate as a perspective material for separation of ^225^Ac and ^213^Bi. J Radioanal Nucl Chem. 2023;332:1475–81.

[15] Mokhodoeva O, Vlk M, Málková E, Kukleva E, Mičolová P, Štamberg K, et al. Study of ^223^Ra uptake mechanism by Fe_3_O_4_ nanoparticles: towards new prospective theranostic SPIONs. J Nanopart Res. 2016;18(10):301.

[16] Zhang J, Zhang S, Cheng C, Zhu C, Wang T, Tang L, et al. Targeting senescence with radioactive ^223^Ra/Ba SAzymes enables senolytics-unlocked one-two punch strategy to boost anti-tumor immunotherapy. Biomaterials. 2025;315:122915. 39461062 10.1016/j.biomaterials.2024.122915

[17] Yang M, Li J, Han Z, Luan X, Zhang X, Gao J, et al. Layered double hydroxides for radium-223 targeted alpha therapy with elicitation of the immune response. Adv Healthc Mater. 2025;14(3):e2403175. 39618118 10.1002/adhm.202403175

[18] Sakmár M, Kozempel J, Kučka J, Janská T, Štíbr M, Vlk M, et al. Biodistribution study of ^211^Pb progeny released from intravenously applied ^223^Ra labelled TiO_2_ nanoparticles in a mouse model. Nucl Med Biol. 2024;130:108890. 38402673 10.1016/j.nucmedbio.2024.108890

[19] Tronchin S, Forster JC, Hickson K, Bezak E. Dosimetry in targeted alpha therapy. A systematic review: current findings and what is needed. Phys Med Biol. 2022;67(9):09TR01. 10.1088/1361-6560/ac5fe0 35316802

[20] De Kruijff RM, Wolterbeek HT, Denkova AG. A critical review of alpha radionuclide therapy-how to deal with recoiling daughters? Pharmaceuticals. 2015;8(2):321–36. 26066613 10.3390/ph8020321 PMC4491664

[21] Cui Z, Wang L, Liu W, Xu D, Zhang T, Ma B, et al. Imageable brachytherapy with chelator-free radiolabeling hydrogel. Adv Healthc Mater. 2024;13(26):e2401438. 38744050 10.1002/adhm.202401438

[22] Chen X, Xia D, Zeng X, Meng L, Wang Y, Li H, et al. Rational design and pharmacomodulation of ^18^F-labeled biotin/FAPI-conjugated heterodimers. J Med Chem. 2024;67(10):8361–71. 38726551 10.1021/acs.jmedchem.4c00544

[23] Lacerda S, De Kruijff RM, Djanashvili K. The advancement of targeted alpha therapy and the role of click chemistry therein. Molecules. 2025;30(6):1296. 40142070 10.3390/molecules30061296 PMC11944744

[24] Tam LKB, Chu JCH, He L, Yang C, Han KC, Cheung PCK, et al. Enzyme-responsive double-locked photodynamic molecular beacon for targeted photodynamic anticancer therapy. J Am Chem Soc. 2023;145(13):7361–75. 36961946 10.1021/jacs.2c13732 PMC10080691

[25] Schauenburg D, Gao B, Rochet LNC, Schüler D, JaS C, Ng DYW, et al. Macrocyclic dual-locked “turn-on” drug for selective and traceless release in cancer cells. Angew Chem Int Ed Engl. 2024;63(18):e202314143. 38179812 10.1002/anie.202314143

[26] Hu Y, Zhang J, Miao Y, Wen X, Wang J, Sun Y, et al. Enzyme-mediated in situ self-assembly promotes in vivo bioorthogonal reaction for pretargeted multimodality imaging. Angew Chem Int Ed Engl. 2021;60(33):18082–93. 34010512 10.1002/anie.202103307

[27] Handula M, Chen KT, Seimbille Y. IEDDA: an attractive bioorthogonal reaction for biomedical applications. Molecules. 2021;26(15):4640. 34361793 10.3390/molecules26154640 PMC8347371

[28] Sun T, Zhang G, Wang Q, Chen Q, Chen X, Lu Y, et al. A targeting theranostics nanomedicine as an alternative approach for hyperthermia perfusion. Biomaterials. 2018;183:268–79. 30179777 10.1016/j.biomaterials.2018.04.016

[29] Chu JCH, Wong CTT, Ng DKP. Toward precise antitumoral photodynamic therapy using a dual receptor-mediated bioorthogonal activation approach. Angew Chem Int Ed Engl. 2023;62(2):e202214473. 36376249 10.1002/anie.202214473

[30] Duan WL, Zhang LN, Bohara R, Martin-Saldaña S, Yang F, Zhao YY, et al. Adhesive hydrogels in osteoarthritis: from design to application. Mil Med Res. 2023;10(1):4. 36710340 10.1186/s40779-022-00439-3 PMC9885614

[31] Fang K, Sun Y, Yang J, Hu X, Chen M, Li R, et al. A dual stimuli-responsive nanoplatform loaded Pt^IV^-triptolide prodrug for achieving synergistic therapy toward breast cancer. Adv Healthc Mater. 2023;12(28):e2301328. 37392128 10.1002/adhm.202301328

[32] Chen X, Zhang S, Li J, Huang X, Ye H, Qiao X, et al. Influence of elasticity of hydrogel nanoparticles on their tumor delivery. Adv Sci. 2022;9(29):e2202644. 10.1002/advs.202202644 PMC9561785 35981891

[33] Hu X, Li R, Wu W, Fang K, Zhu Z, Wang Y, et al. A Fe(III)-porphyrin-oxaliplatin(IV) nanoplatform for enhanced ferroptosis and combined therapy. J Control Release. 2022;348:660–71. 35716884 10.1016/j.jconrel.2022.06.019

[34] Navarro G, Gómez-Autet M, Morales P, Rebassa JB, Llinas Del Torrent C, Jagerovic N, et al. Homodimerization of CB2 cannabinoid receptor triggered by a bivalent ligand enhances cellular signaling. Pharmacol Res. 2024;208:107363. 39179054 10.1016/j.phrs.2024.107363

[35] Yang X, Xu C, Zeng Y, Wang C, Gao Y, Ding J, et al. Pyroptosis-inducing platinum^IV^ prodrugs via GSDME pathway for chemoimmunotherapy and metastasis inhibition in triple-negative breast cancer. Adv Sci. 2025;12(29):e05567. 10.1002/advs.202505567 PMC12362741 40432601

[36] Yang X, Huang N, Li M, Zhu X, Wang C, Zhang Y, et al. A new oxaliplatin^IV^ prodrug inducing ferroptosis through a triple-pathway mechanism and activating anti-cancer immunity. Sci China Chem. 2025;68(9):4297–310.

[37] Engeland K. Cell cycle regulation: p53–p21-RB signaling. Cell Death Differ. 2022;29(5):946–60. 35361964 10.1038/s41418-022-00988-z PMC9090780

[38] Fischer M, Quaas M, Steiner L, Engeland K. The p53–p21-DREAM-CDE/CHR pathway regulates G2/M cell cycle genes. Nucleic Acids Res. 2016;44(1):164–74. 26384566 10.1093/nar/gkv927 PMC4705690

[39] Qi R, Wang J, Jiang Y, Qiu Y, Xu M, Rong R, et al. Snai1-induced partial epithelial-mesenchymal transition orchestrates p53–p21-mediated G2/M arrest in the progression of renal fibrosis via NF-κB-mediated inflammation. Cell Death Dis. 2021;12(1):44. 33414422 10.1038/s41419-020-03322-y PMC7790819

[40] Krysko DV, Garg AD, Kaczmarek A, Krysko O, Agostinis P, Vandenabeele P. Immunogenic cell death and DAMPs in cancer therapy. Nat Rev Cancer. 2012;12(12):860–75. 23151605 10.1038/nrc3380

[41] Mandula JK, Chang S, Mohamed E, Jimenez R, Sierra-Mondragon RA, Chang DC, et al. Ablation of the endoplasmic reticulum stress kinase PERK induces paraptosis and type I interferon to promote anti-tumor T cell responses. Cancer Cell. 2022;40(10):1145-60.e9. 36150390 10.1016/j.ccell.2022.08.016 PMC9561067

[42] Guo Y, Bao Q, Hu P, Shi J. Nanomedicine-based co-delivery of a calcium channel inhibitor and a small molecule targeting CD47 for lung cancer immunotherapy. Nat Commun. 2023;14(1):7306. 37951973 10.1038/s41467-023-42972-2 PMC10640620

[43] Hsieh RC, Krishnan S, Wu RC, Boda AR, Liu A, Winkler M, et al. ATR-mediated CD47 and PD-L1 up-regulation restricts radiotherapy-induced immune priming and abscopal responses in colorectal cancer. Sci Immunol. 2022;7(72):eabl9330. 35687697 10.1126/sciimmunol.abl9330 PMC9373855

[44] Wen ZQ, Lin J, Xie WQ, Shan YH, Zhen GH, Li YS. Insights into the underlying pathogenesis and therapeutic potential of endoplasmic reticulum stress in degenerative musculoskeletal diseases. Mil Med Res. 2023;10(1):54. 37941072 10.1186/s40779-023-00485-5 PMC10634069

[45] Shang K, Montesdeoca N, Zhang H, Efanova E, Liang G, Ochs J, et al. Cobalt^III^ prodrug-based nanomedicine for inducing immunogenic cell death and enhancing chemo-immunotherapy. J Control Release. 2024;373:493–506. 39033985 10.1016/j.jconrel.2024.07.042

[46] Yuan Y, Jiao P, Wang Z, Chen M, Du H, Xu L, et al. Endoplasmic reticulum stress promotes the release of exosomal PD-L1 from head and neck cancer cells and facilitates M2 macrophage polarization. Cell Commun Signal. 2022;20(1):12. 35090495 10.1186/s12964-021-00810-2 PMC8796490

[47] Ketkar M, Desai S, Rana P, Thorat R, Epari S, Dutt A, et al. Inhibition of PERK-mediated unfolded protein response acts as a switch for reversal of residual senescence and as senolytic therapy in glioblastoma. Neuro Oncol. 2024;26(11):2027–43. 39021199 10.1093/neuonc/noae134 PMC11534322

[48] Chauvin M, Borys D, Botta F, Bzowski P, Dabin J, Denis-Bacelar AM, et al. OpenDose: open-access resource for nuclear medicine dosimetry. J Nucl Med. 2020;61(10):1514–9. 32169912 10.2967/jnumed.119.240366 PMC7539649

[49] Talaat K, Xi J, Baldez P, Hecht A. Radiation dosimetry of inhaled radioactive aerosols: CFPD and MCNP transport simulations of radionuclides in the lung. Sci Rep. 2019;9(1):17450. 31768010 10.1038/s41598-019-54040-1 PMC6877642

[50] Elbast M, Saudo A, Franck D, Petitot F, Desbrée A. Microdosimetry of alpha particles for simple and 3D voxelised geometries using MCNPX and Geant4 Monte Carlo codes. Radiat Prot Dosim. 2012;150(3):342–9. 10.1093/rpd/ncr401. 10.1093/rpd/ncr401 21993801

[51] Botta F, Mairani A, Battistoni G, Cremonesi M, Di Dia A, Fassò A, et al. Calculation of electron and isotopes dose point kernels with FLUKA Monte Carlo code for dosimetry in nuclear medicine therapy. Med Phys. 2011;38(7):3944–54. 21858991 10.1118/1.3586038

