# Supplementary material for: Dual-locked targeted alpha-emitter enhanced tumor immunotherapy via Diels–Alder reaction-based self-immolative molecular cage strategy
Source: Mil Med Res. 2025 Dec 1;12:84. doi: 10.1186/s40779-025-00673-5 (PMC12667170; doi:10.1186/s40779-025-00673-5)
Supplement: Supplementary file 1 — Additional file 1. Methods. Fig. S1 ESI-MS spectrum of PtIV. Fig. S2 1H-NMR spectrum of HA (a) and HAQ (b) in D2O. Fig. S3 TEM-elemental mapping of HAQ@HNPs. Fig. S4 The stability of the HAQ@HNPs incubated in different media. Fig. S5 Characterization of compound 2. Fig. S6 Characterization of compound 3. Fig. S7 Characterization of compound 4. Fig. S8 Characterization of compound 5. Fig. S9 Characterization of compound 6. Fig. S10 Characterization of compound 7. Fig. S11 1H-NMR spectrum of self-immolating spacer in methanol-d4. Fig. S12 DLS result of HNPs-NH2 and Ba-HNPs. Fig. S13 The release of Ba2+ and PtIV from nanogel. Fig. S14 Cytotoxicity of HAQ/223Ra@HNPs in vitro. Fig. S15 Biodistribution profile. Fig. S16 Representative DAR and H&E images of frozen tissue sections from B16/F10-bearing mice following different treatments at 72 h post-injection. Fig. S17 Time-activity curves and fitted functions for biodistribution of 223Ra and HAQ/223Ra@HNPs. Fig. S18 Ex vivo tumor weights from each group at day 10 (n = 5) in B16/F10 tumor-bearing mice. Fig. S19 Antitumor evaluations of HAQ/223Ra@HNPs in LLC tumor-bearing mice. Fig. S20 Representative histological images of the main organs in B16/F10-bearing mice after different treatments, stained with hematoxylin and eosin. Fig. S21 Hematological analysis was performed on blood withdrawn from B16/F10 tumor-bearing mice in corresponding treatment groups at the terminal of study (n = 5). Fig. S22 Immunofluorescence staining of p53 and p21 in tumor tissues following different treatments. Fig. S23 Immunofluorescence staining of GRP78 and PERK in tumor tissues following different treatments. Fig. S24 Immunofluorescence staining of PD-L1 in tumor tissues following different treatments. Fig. S25 Representative flow cytometry plots showing DCs maturation in the spleen, DCs maturation, CD4+ T cells, and CD8+ T cells in tumors from different groups (n = 3). Fig. S26 The secretion of cytokines TNF-α and IFN-γ. Fig. S27 Ex vivo tumor [file 40779_2025_673_MOESM1_ESM.pdf]

Methods

Materials and instruments

All the reagents were utilized without further purification. Unless otherwise stated, all chemical reagents were obtained from Tansoole (China). ^{1}H-nuclear magnetic resonance (^{1}H-NMR) and ^{13}C-NMR were tested by Bruker 400 MHz NMR spectrometer (Bruker, Germany). Thermo Scientific Q Exactive combined quadrupole Orbitrap mass spectrometer (Thermo Fisher Scientific, USA) was used to obtain high-resolution mass spectrometry (HRMS) spectra. Transmission electron microscopy (TEM) images were obtained through JEOL JEM-2100 transmission electron microscope (JEOL, Japan). The content of platinum was measured by inductively coupled plasma optical emission spectrometer (Optima 8300 ICP-OES, PerkinElmer, USA). Dynamic light scattering (DLS) and Zeta-potential were determined by Malvern Zetasizer Nano ZS instrument (Malvern Instruments, UK). Ultraviolet (UV)-visible spectra analysis was determined by Hitachi U-2900 Spectrophotometer (Hitachi, Japan). Digital autoradiography (DAR) was obtained by Amersham Typhoon IP (Cat. No. 29187194; Cytiva, USA).

Synthesis of compound 2

Imidazole and tert-butylchlorodimethylsilane chloride (TBSCl) were dissolved in anhydrous dichloromethane (CH_2Cl_2) and subsequently injected into a solution of (2-hydroxy-5-methyl-1,3-phenylene) dimethanol in anhydrous N,N-dimethylformamide (DMF). The resulting mixture was allowed to react at room temperature for 4 h. Following the reaction, the mixture was washed successively with water and brine, dried over anhydrous sodium sulfate, and purified by column chromatography, yielding compound 2 (yield > 90%) as an oily liquid. ^{1}H-NMR [400 MHz, deuterated chloroform ($CDCl_3$), unit: ppm] δ8.05 (s, 1H, H1), 6.93 (s, 2H, H2 – H3), 4.85 (s, 4H, H4 – H7), 2.28 (s, 3H, H8 – H10), 0.97 (s, 18H, H11 – H28), 0.15 (s, 12H, H29 – H40). ^{13}C-NMR (400 MHz, $CDCl_3$) δ 151.06, 128.42, 126.33, 125.92, 63.17, 26.04, 20.82, 18.46, –5.26. ESI-MS: *m/z* $[M-H]^-$ calculated for $C_{21}H_{39}O_3Si_2$: 395.2438, found 395.2442.

Synthesis of compound 3

Compound 2 and N,N-diisopropylethylamine (DIPEA) were dissolved in anhydrous CH_2Cl_2, which was then cooled to 0 °C. Subsequently, 4-nitrophenyl carbonochloridate in anhydrous CH_2Cl_2 was injected dropwise into the aforementioned solution. The mixture was allowed to reach room

temperature and was maintained at this temperature for 4 h. After the reaction, the mixture was washed successively with water and brine, dried over anhydrous sodium sulfate, and purified by column chromatography, yielding compound 2 (yield > 90%) as a white solid. ^{1}H-NMR (400 MHz, $CDCl_3$, unit: ppm) δ 8.31 (d, J = 9.2 Hz, 2H, H1 – H2), 7.48 (d, J = 9.1 Hz, 2H, H4 – H5), 7.22 (s, 2H, H6-H7), 4.72 (s, 4H, H8 – H11), 2.38 (s, 3H, H12 – H14), 0.93 (s, 18H, H15 – H32), 0.09 (s, 12H, H33 – H44). ^{13}C-NMR (400 MHz, $CDCl_3$) δ 155.73, 150.26, 145.63, 143.48, 136.80, 132.86, 128.11, 125.52, 121.57, 60.73, 31.66, 31.59, 30.36, 30.30, 29.84, 26.07, 21.34, 18.59. ESI-MS: *m/z* $[M+H]^+$ calculated for $C_{28}H_{44}NO_7Si_2$: 562.2656, found 562.2641.

Synthesis of compound 4

Bicyclo[6.1.0]nonyne (BCN) and triethylamine (TEA) were injected into compound 3 in anhydrous DMF using a syringe over a period of 15 min. The resulting mixture was allowed to equilibrate to room temperature and maintained for 12 h. Subsequently, the reaction mixture was diluted with ethyl acetate, washed successively with water and brine, dried over anhydrous sodium sulfate, and purified by column chromatography (CH_2Cl_2/CH_3OH, 20:1) to yield compound 4. ^{1}H-NMR (400 MHz, $CDCl_3$, unit: ppm) δ 8.59 (d, J = 8.1 Hz, 2H, H4 – H5), 7.56 (d, J = 8.1 Hz, 2H, H6 – H7), 7.21 (s, 2H, H11 – H12), 5.52 (t, 1H, H10) 4.66 (s, 4H, H13 – H16), 4.57 (d, J = 6.3 Hz, 2H, H8 – H9), 3.11 (s, 3H, H1 – H3), 2.35 (s, 3H, H17 – H19), 0.93 (s, 18H, H20 – H37), 0.08 (s, 12H, H38 – H49). ^{13}C-NMR (400 MHz, $CDCl_3$) δ 167.46, 163.97, 162.15, 154.42, 143.21, 142.40, 135.84, 133.60, 131.36, 128.52, 128.26, 127.15, 126.17, 115.71, 77.37, 77.16, 77.15, 76.95, 60.25, 45.15, 26.09, 25.78, 21.44, 21.30, 18.58. ESI-MS: *m/z* $[M+H]^+$ calculated for $C_{32}H_{50}N_5O_4Si_2$: 624.3401, found 624.3398.

Synthesis of compound 5

Compound 5 and ρ-toluenesulfonic acid were suspended in methanol and allowed to react at room temperature for 4 h. Subsequently, the solvent was removed using a rotary evaporator, and the reaction mixture was diluted with ethyl acetate. The mixture was then washed successively with water and brine, dried over anhydrous sodium sulfate, and purified by column chromatography using a solvent system of CH_2Cl_2/CH_3OH in a 20:1 ratio to yield compound 5. ^{1}H-NMR (400 MHz, DMSO-d_6, unit: ppm) δ 8.47 (d, J = 8.0 Hz, 2H, H4 – H5), 7.60 (d, J = 8.0 Hz, 2H, H6 – H7), 7.17 (s, 2H, H11 – H12), 5.12 (d, J = 5.6 Hz, 1H, H10), 4.41 (t, J = 5.5 Hz, 8H, H8, 9, 13, 14, 15, 16, 17, 18), 3.00 (s, 3H, H1 – H3), 2.31 (s, 3H, H19 – H21). ^{13}C-NMR (400 MHz, DMSO-d_6) δ 167.06, 163.17, 154.47, 144.24, 142.53, 134.61, 134.02, 130.54, 127.74, 127.51, 126.10, 57.72, 54.86, 48.58, 43.76, 20.81, 20.79. ESI-MS: *m/z*

[M-H_2O+H]$^+$ calculated for $C_{20}H_{20}N_5O_3$: 378.1566, found 378.1562.

Synthesis of compound 6

A solution of compound 5 in pyridine was treated with 4-dimethylaminopyridine (DMAP) and succinic anhydride. After allowing the reaction to proceed overnight under argon protection, the mixture was diluted with ethyl acetate and subsequently washed 3 times with saturated sodium chloride solution. The ethyl acetate layers were then dried over Na_2SO_4 and purified by silica gel column chromatography using a solvent system of CH_2Cl_2/CH_3OH (15:1), yielding compound 6 (yield > 80%) as a red powder. ^{1}H-NMR (400 MHz, $CDCl_3$, unit: ppm) δ 8.46 (d, J = 8.0 Hz, 2H, H4 – H5), 7.60 (d, J = 8.0 Hz, 2H, H6 – H7), 7.28 (d, J = 2.3 Hz, 1H, H12), 7.11 (d, J = 2.3 Hz, 1H, H11), 5.20 (m, 1H, H10), 4.99 (s, 2H, H15 – H16), 4.57 – 4.24 (m, 5H, H8 – H9, H13 – H14, H17), 3.00 (s, 3H, H1 – H3), 2.49 – 2.41 (m, 4H, H18 – H21), 2.31 (s, 3H, H22-H24). ^{13}C-NMR (400 MHz, $CDCl_3$) δ 172.00, 167.07, 163.17, 154.34, 144.08, 143.72, 135.45, 134.53, 130.57, 128.41, 128.05, 127.87, 127.75, 127.52, 60.99, 57.61, 43.79, 28.89, 28.78, 20.81, 20.61. ESI-MS: m/z [M+H]$^+$ calculated for $C_{24}H_{26}N_5O_7$: 496.1832, found 496.1810.

Synthesis of compound 7

Compound 6 and DIPEA were dissolved in anhydrous CH_2Cl_2, which was then cooled to 0 °C. Subsequently, 4-nitrophenyl carbonochloridate in anhydrous CH_2Cl_2 was injected dropwise into the aforementioned solution. The mixture was allowed to reach room temperature and was maintained at this temperature for 4 h. After the reaction, the mixture was washed successively with water and brine, dried over anhydrous sodium sulfate, and purified by column chromatography, yielding compound 2 (yield > 90%) as a red solid. ^{1}H-NMR (400 MHz, $CDCl_3$, unit: ppm) δ 8.54 (d, J = 8.0 Hz, 2H, H4 – H5), 8.16 (d, J = 8.6 Hz, 2H, H15 – H16), 7.50 (d, J = 8.0 Hz, 2H, H6 – H7), 7.30 – 7.16 (m, 4H, H11 – H14), 6.01 (t, J = 6.3 Hz, 1H, H10), 5.13 (s, 2H, H17 – H18), 4.54 (s, 2H, H19 – H20), 4.49 (d, J = 6.2 Hz, 2H, H8 – H9), 3.09 (s, 3H, H1 – H3), 2.89 (m, 2H, H21 – H22), 2.71 (m, 2H, H23 – H24), 2.33 (s, 3H, H25 – H27). ^{13}C-NMR (400 MHz, $CDCl_3$) δ 171.75, 170.60, 167.47, 163.93, 155.56, 155.25, 145.50, 145.35, 142.74, 136.58, 134.23, 131.74, 131.36, 131.13, 128.46, 128.36, 128.06, 125.27, 122.44, 62.64, 60.47, 45.14, 29.82, 29.35, 28.90, 21.28, 20.93. ESI-MS: m/z [M-CO_2+H]$^+$ calculated for $C_{30}H_{29}N_6O_9$: 617.1996, found 617.1994.

Cells culture

The B16/F10 cell lines were sourced from Hai Baina Biotechnology Co., Ltd. (Shandong, China). Cells were cultured in RPMI 1640 medium supplemented with 10% fetal bovine serum (FBS) and 1% penicillin-streptomycin at 37 °C under an atmosphere of 5% CO_2 and 95% air (approximately 20% O_2).

Cell cycle analysis

To assess the effects of treatment on cell cycle distribution, B16/F10 cells were seeded in 6-well plates at a density of 5×10^5 cells per well and allowed to adhere overnight. Cells were then treated with Pt^{IV}, free ^{223}Ra, HAQ@HNPs, ^{223}Ra@HNPs, HAQ/^{223}Ra@HNPs, or control for 24 h under standard culture conditions (37 °C, 5% CO_2). After treatment, cells were harvested by trypsinization, washed twice with cold phosphate-buffered saline (PBS), and fixed in 70% ice-cold ethanol overnight at –20 °C. The fixed cells were then centrifuged at 1000 × *g* for 5 min, washed with PBS, and resuspended in 500 μl of propidium iodide (PI) staining solution containing 50 μg/ml PI and 100 μg/ml RNase A. The cell suspension was incubated at 37 °C in the dark for 30 min to allow DNA staining. Cell cycle distribution was analyzed using a flow cytometer by measuring PI fluorescence at an excitation wavelength of 488 nm. The percentage of cells in G0/G1, S, and G2/M phases was determined using FlowJo software.

In vitro apoptosis assay

B16/F10 cells (5×10^5 cells/well) were seeded in 6-well plates and incubated overnight. Cells were treated under the same conditions as in the Cell Cycle Analysis. After 24 h of treatment, cells were stained with Annexin V/PI and analyzed via flow cytometry to determine apoptotic cell populations.

Intracellular reactive oxygen species (ROS) detection

To assess oxidative stress, B16/F10 cells were treated as previously described and incubated with 2′,7′-dichlorodihydrofluorescein diacetate (DCFH-DA) for 30 min. Intracellular ROS generation was quantified by flow cytometry.

Intracellular glutathione (GSH) levels detection

To evaluate the depletion of intracellular GSH, B16/F10 cells were treated with the same conditions as above for 24 h. GSH and GSSG levels were quantified using an assay kit following the manufacturer's protocol. Absorbance at 412 nm was measured with a microplate reader. Reduced GSH levels were determined by subtracting twice the GSSG concentration from total GSH.

In vivo imaging study

Tumor-bearing C57BL/6 mice received an intra-tumoral injection when the tumor diameter reached approximately 100 mm^3, and fluorescence imaging of mice was conducted at different time points (6, 24 h) after intra-tumoral injection with HAQ/Cy7-^{223}Ra@HNPs using the infrared imaging system. The pretargeted strategy involved intratumoral administration of HAQ@HNPs (10 μg Pt^{IV}), followed by intravenous injection of ^{223}Ra@HNPs (9.25 kBq).

Immunofluorescence staining

After 10 d of treatment, tumor tissues of mice were collected and used to make immunofluorescence staining analysis. the variation of p53, p21, glucose-regulated protein 78 (GRP78), PKR-like endoplasmic reticulum kinase (PERK), and PD-L1 expression in different tumor tissues was examined by immunofluorescence staining with the corresponding antibody.

RNA sequencing (RNA-seq) analysis

When the tumor grew to 50 mm^3, the tumor-bearing mice were randomly divided into 2 groups (n = 3). The control group received 100 μl of PBS buffer, while the treatment group was administered HAQ/^{223}Ra@HNPs. Tumor tissues were harvested from mice 7 d after resection for RNA-seq. RNA extraction, quality control, library construction, and sequencing were performed according to the company's standard procedures. The featureCounts and DESeq2software were used to quantify gene expression and perform differential expression analysis between the two comparison groups, respectively. Gene Ontology (GO) enrichment analysis and gene set enrichment analysis (GSEA) analysis were conducted using clusterProfiler.

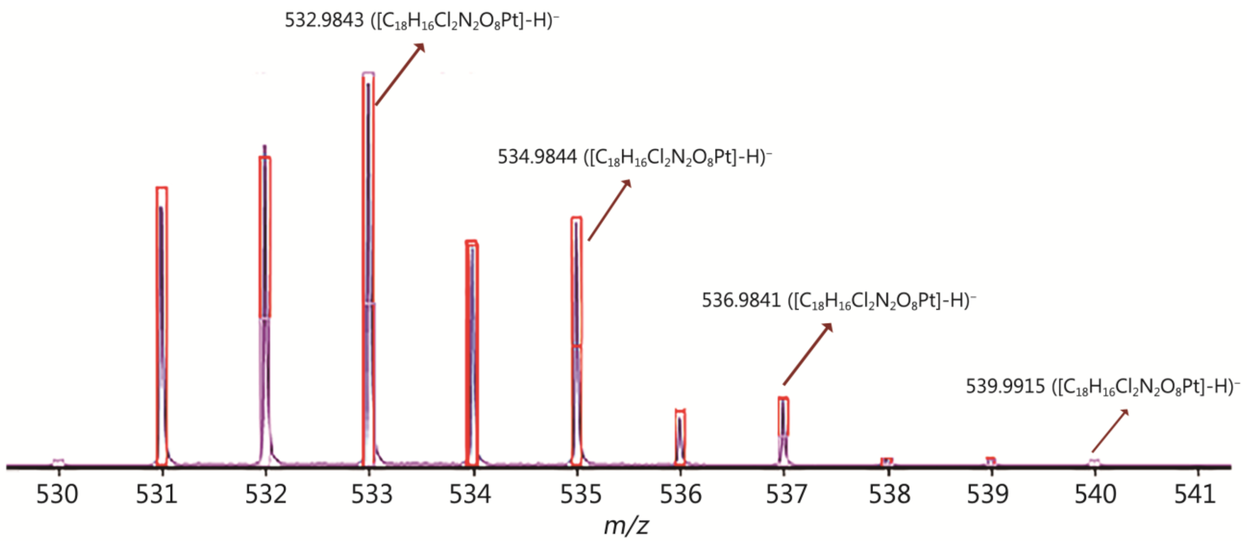

Fig. S1 ESI-MS spectrum of Pt^{IV}. ESI-MS electrospray ionization mass spectrometry, Pt^{IV} platinum^{IV}

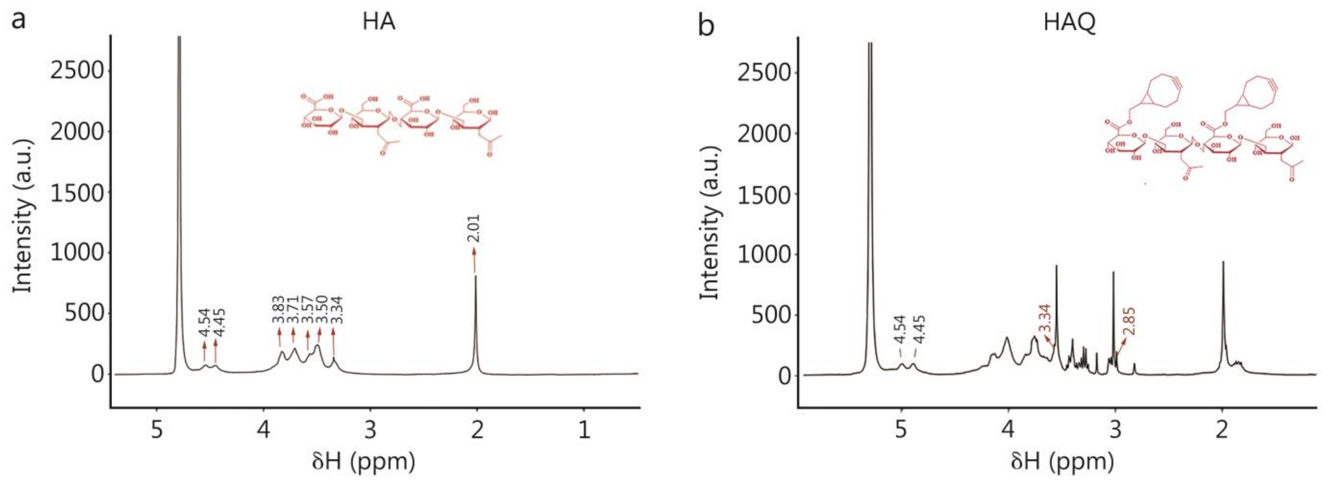

Fig. S2 ^{1}H-NMR spectrum of HA (**a**) and HAQ (**b**) in D_2O. ^{1}H-NMR ^{1}H-nuclear magnetic resonance, D_2O deuterium oxide, δH ^{1}H-NMR chemical shift, HAQ hyaluronic acid (HA) with bicyclo[6.1.0]nonyne (BCN)

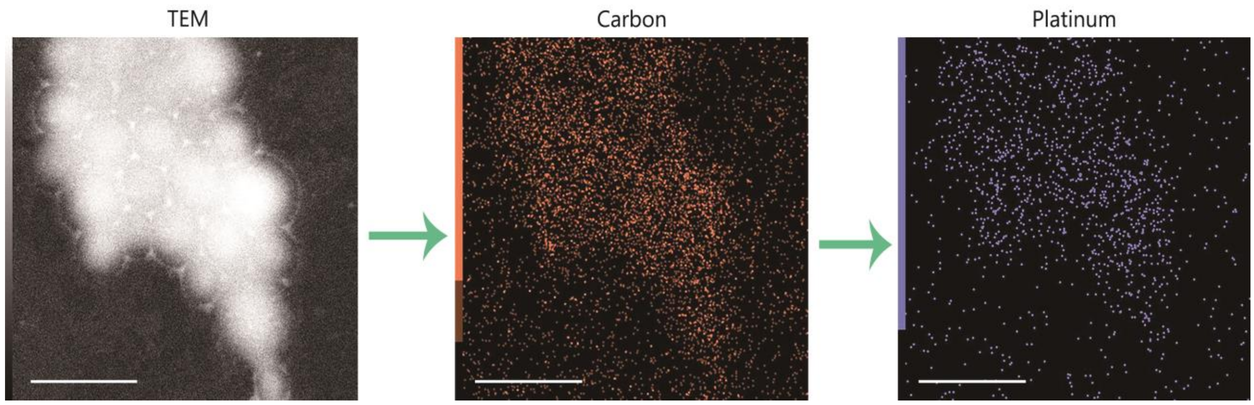

Fig. S3 TEM-elemental mapping of HAQ@HNPs. Scale bar = 500 nm. TEM transmission electron microscopy, HAQ@HNPs platinum^{IV}-loaded hydrogel nanoparticles with HAQ, HAQ hyaluronic acid (HA) with bicyclo[6.1.0]nonyne (BCN)

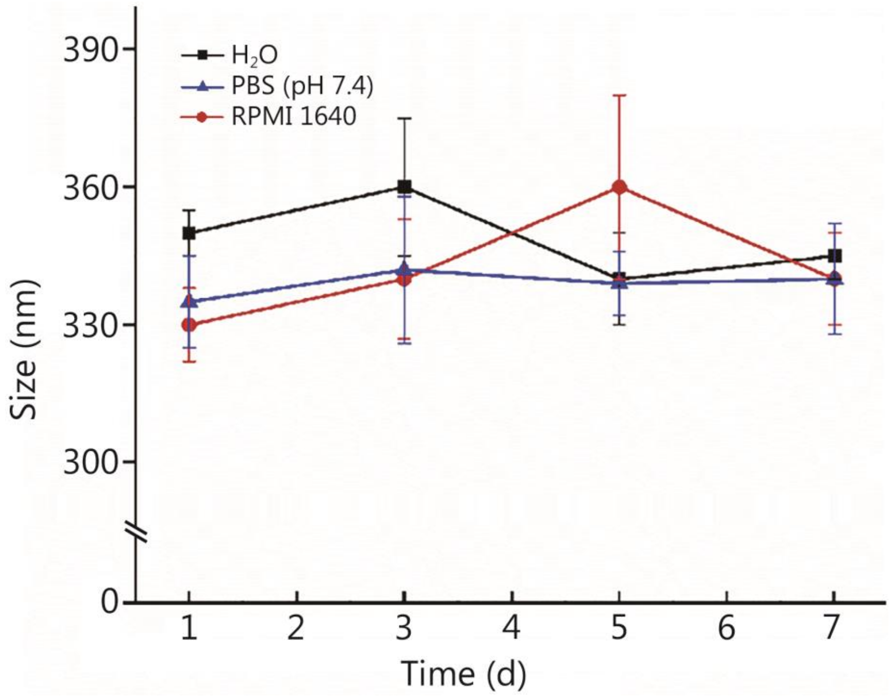

Fig. S4 The stability of the HAQ@HNPs incubated in different media. PBS phosphate-buffered saline, HAQ@HNPs Pt^{IV}-loaded hydrogel nanoparticles with HAQ, HAQ hyaluronic acid (HA) with bicyclo[6.1.0]nonyne (BCN)

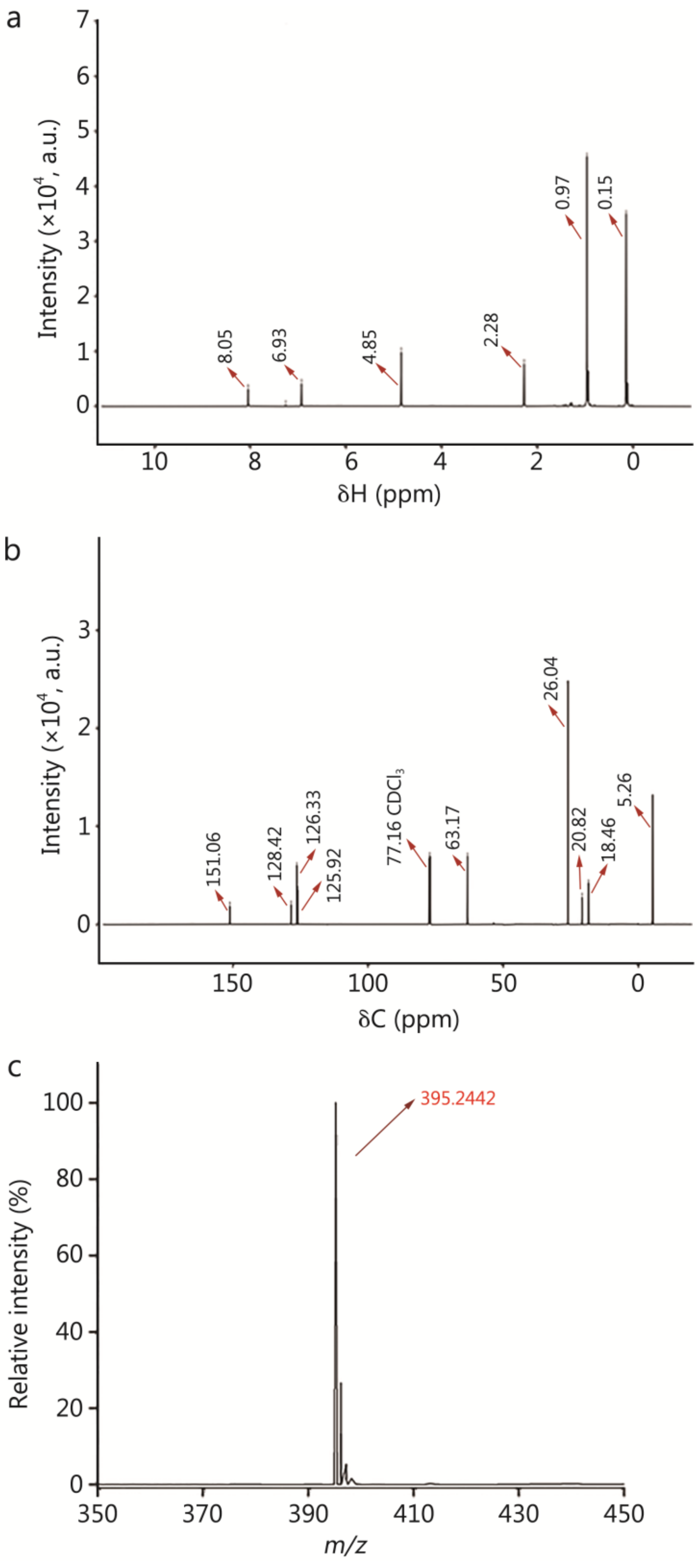

Fig. S5 Characterization of compound 2. ^{1}H-NMR (**a**) and ^{13}C-NMR (**b**) spectrum of compound 2 in $CDCl_3$. **c** ESI-MS of compound 2, negative mode in methanol. NMR nuclear magnetic resonance, ESI-MS electrospray ionization mass spectrometry, δH ^{1}H-NMR chemical shift, δC ^{13}C-NMR chemical shift, $CDCl_3$ deuterated chloroform

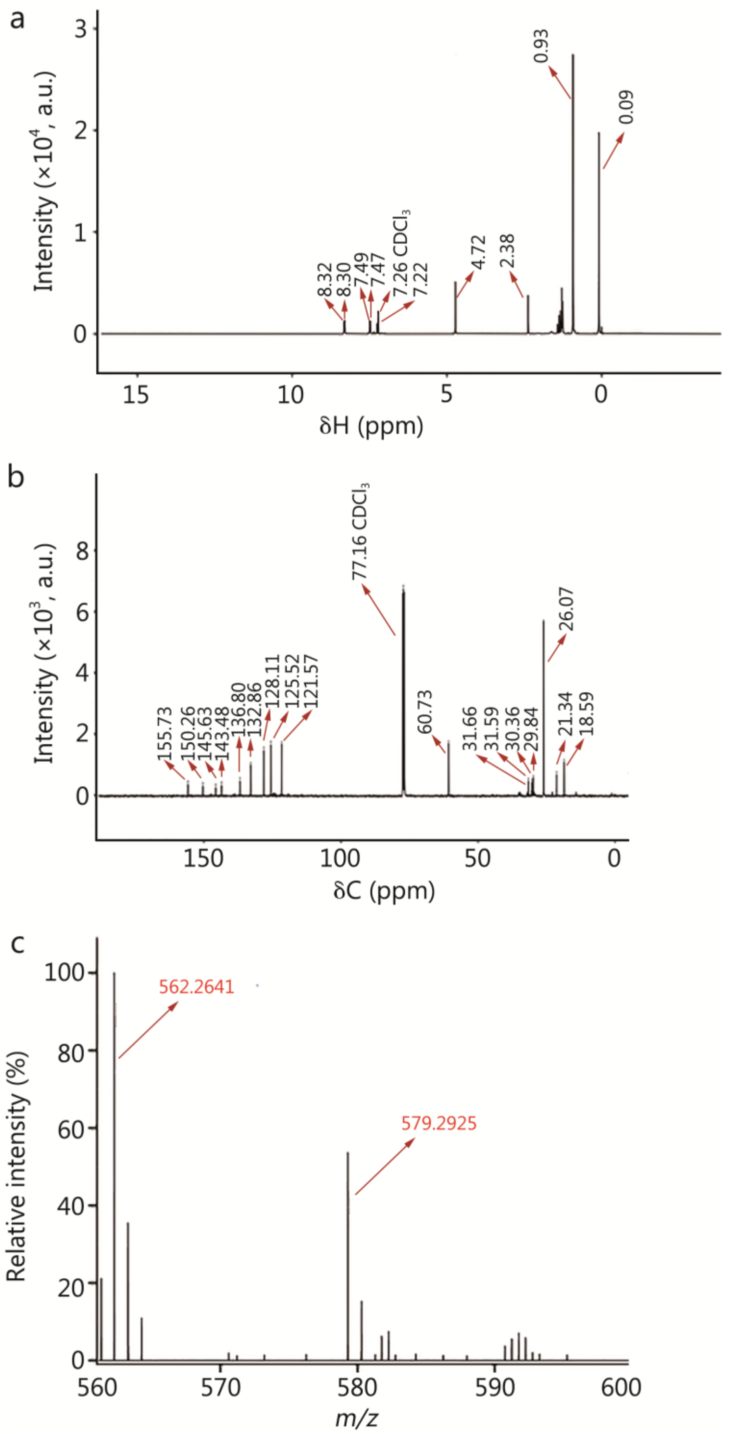

Fig. S6 Characterization of compound 3. ^{1}H-NMR (**a**) and ^{13}C-NMR (**b**) spectrum of compound 3 in $CDCl_3$. **c** ESI-MS of compound 3, positive mode in methanol. NMR nuclear magnetic resonance, ESI-MS electrospray ionization mass spectrometry, δH ^{1}H-NMR chemical shift, δC ^{13}C-NMR chemical shift, $CDCl_3$ deuterated chloroform

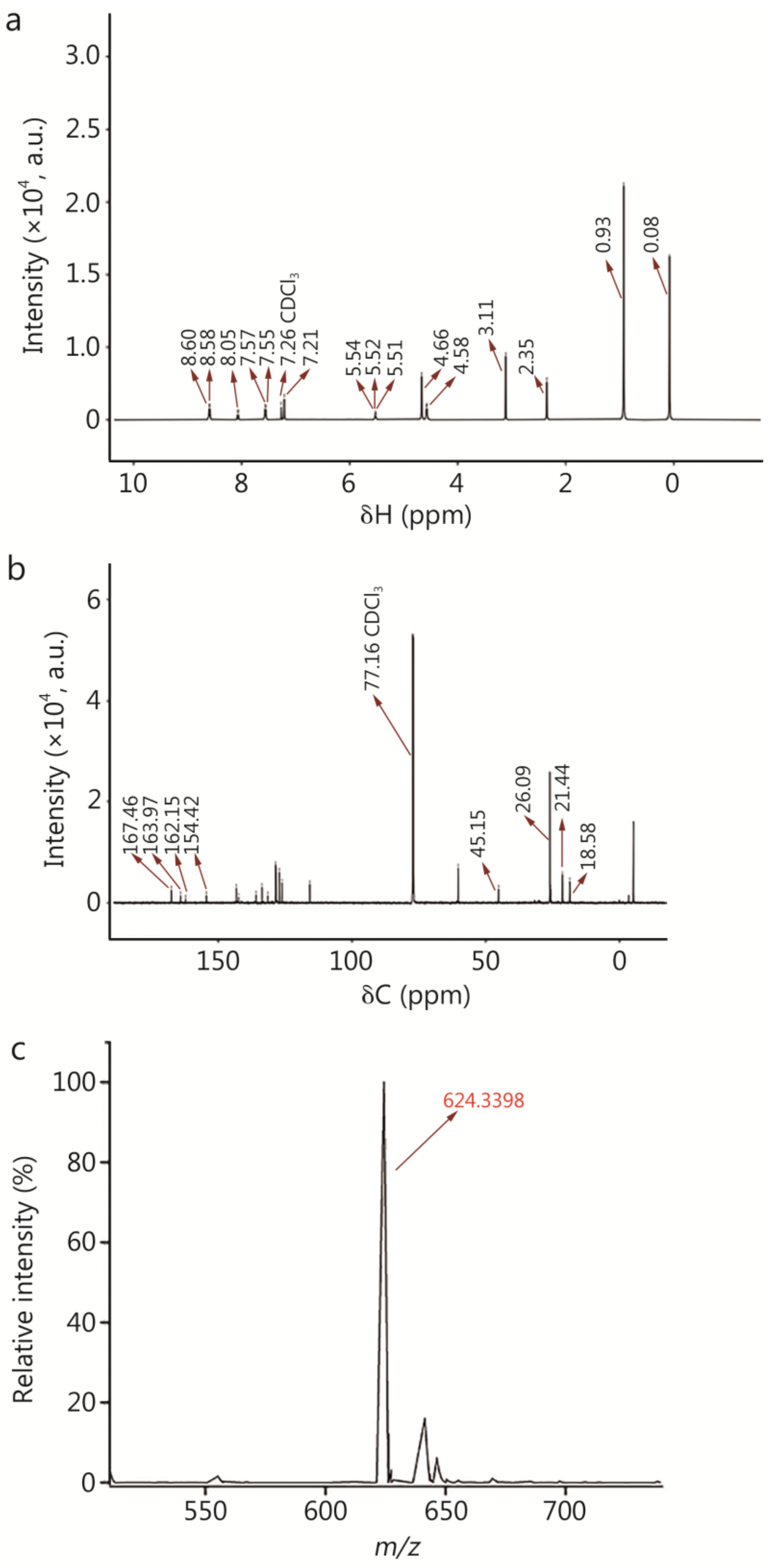

Fig. S7 Characterization of compound 4. ^{1}H-NMR (**a**) and ^{13}C-NMR (**b**) spectrum of compound 4 in $CDCl_3$. **c** ESI-MS of compound 4, positive mode in methanol. NMR nuclear magnetic resonance, ESI-MS electrospray ionization mass spectrometry, δH ^{1}H-NMR chemical shift, δC ^{13}C-NMR chemical shift, $CDCl_3$ deuterated chloroform

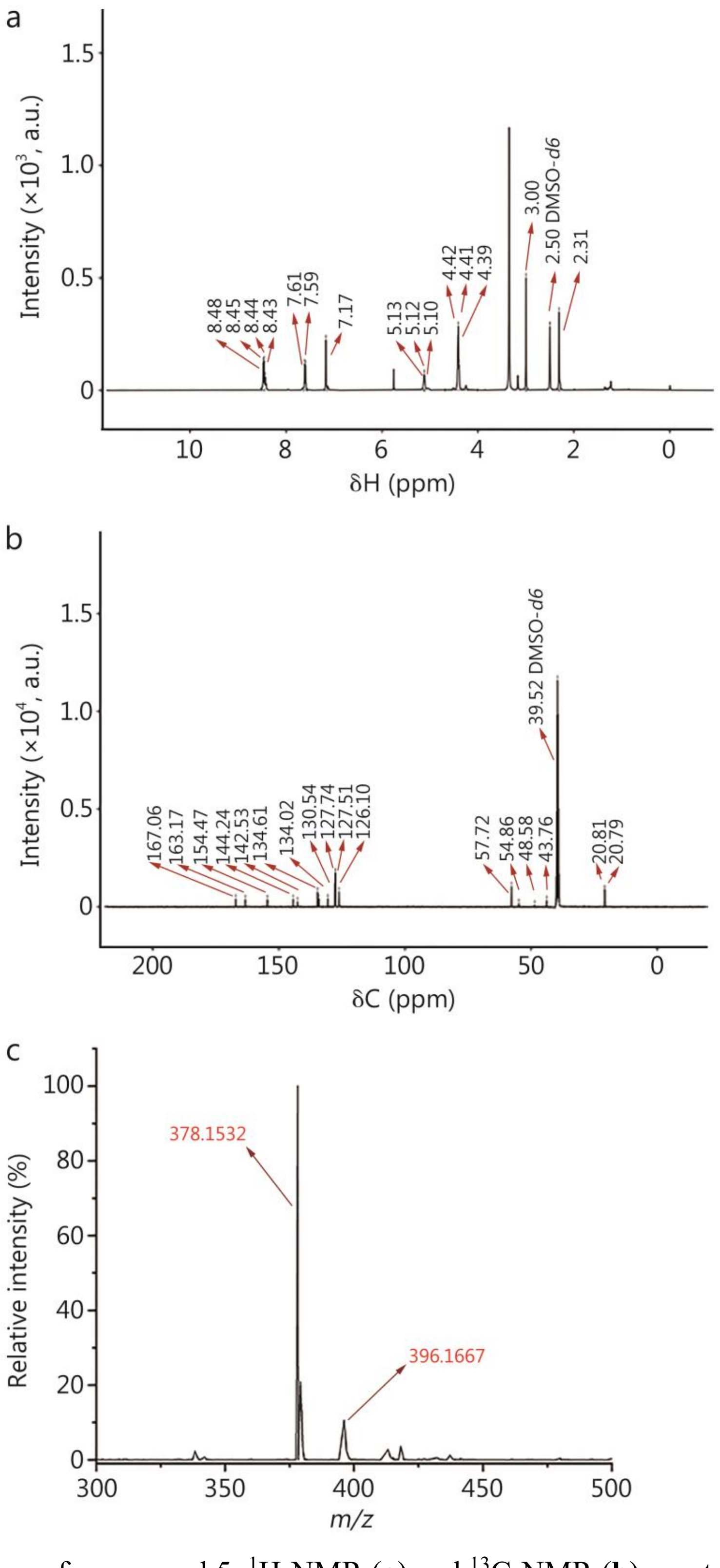

Fig. S8 Characterization of compound 5. ^{1}H-NMR (**a**) and ^{13}C-NMR (**b**) spectrum of compound 5 in DMSO-*d6*. **c** ESI-MS of compound 5, positive mode in methanol. NMR nuclear magnetic resonance, ESI-MS electrospray ionization mass spectrometry, ESI-MS electrospray ionization mass spectrometry, δH ^{1}H-NMR chemical shift, δC ^{13}C-NMR chemical shift, DMSO-*d6* deuterated dimethyl sulfoxide

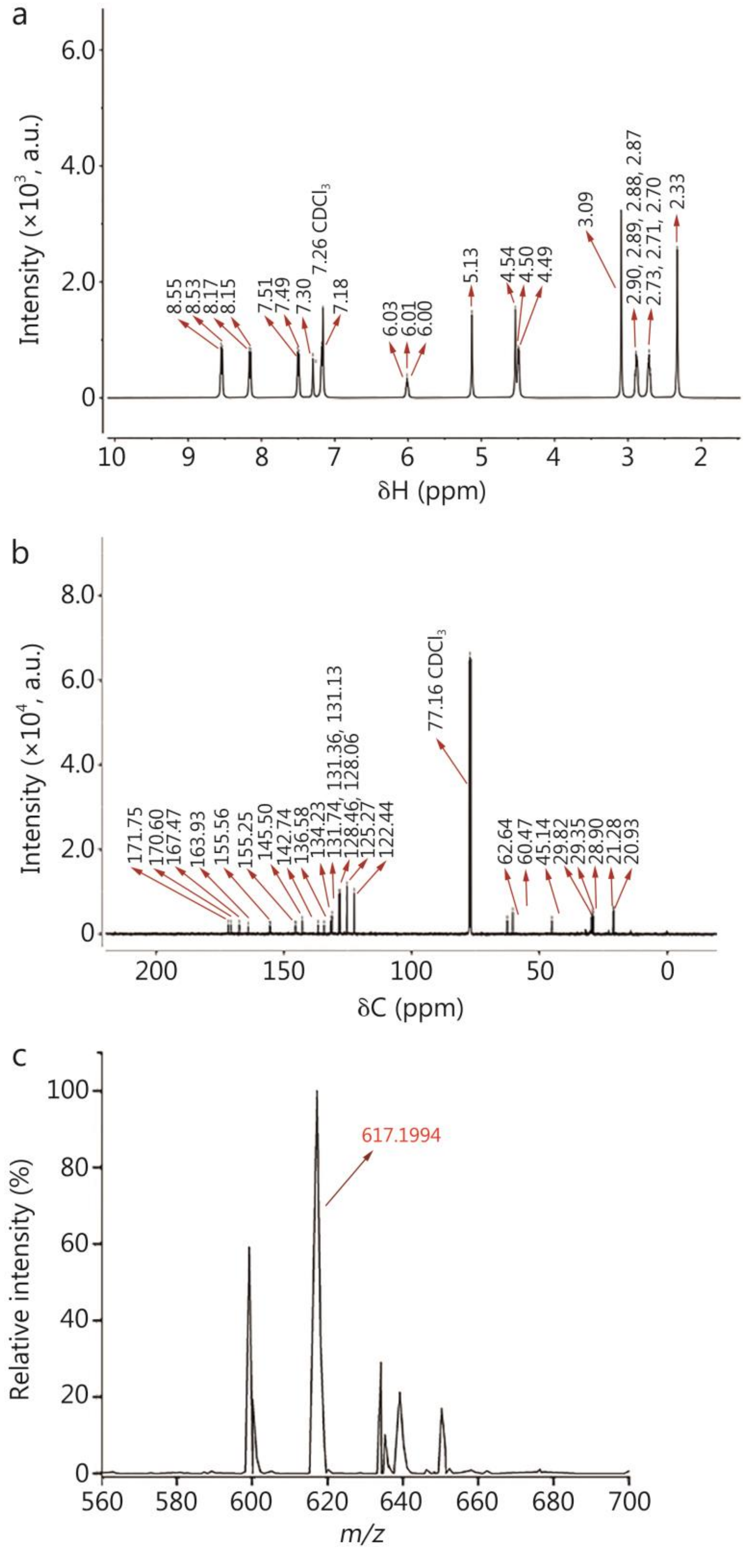

Fig. S9 Characterization of compound 6. ^{1}H-NMR (**a**) and ^{13}C-NMR (**b**) spectrum of compound 6 in $CDCl_3$. **c** ESI-MS of compound 6, positive mode in methanol. NMR nuclear magnetic resonance, ESI-MS electrospray ionization mass spectrometry, δH ^{1}H-NMR chemical shift, δC ^{13}C-NMR chemical shift, $CDCl_3$ deuterated chloroform

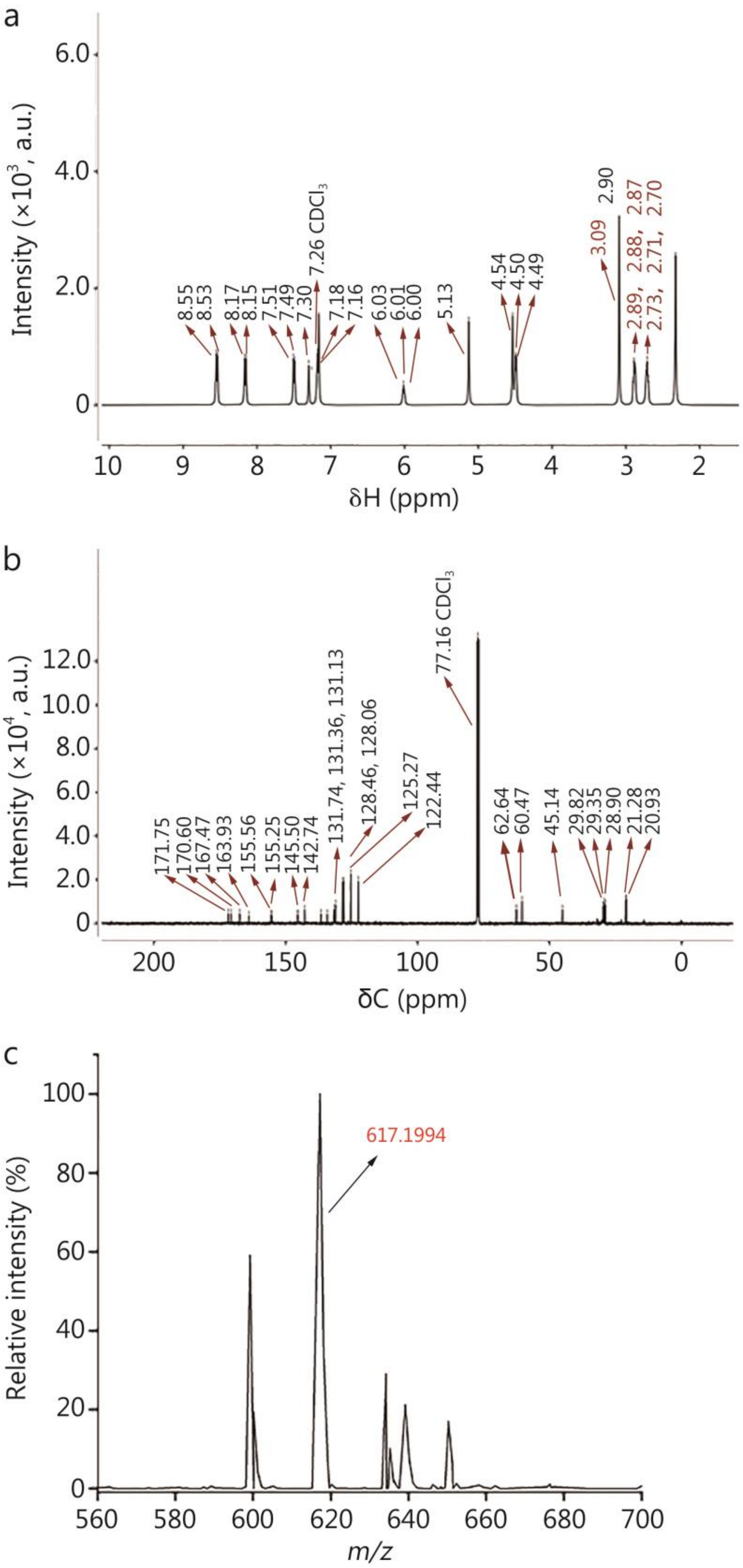

Fig. S10 Characterization of compound 7. ^{1}H-NMR (**a**) and ^{13}C-NMR (**b**) spectrum of compound 7 in $CDCl_3$. **c** ESI-MS of compound 7, positive mode in methanol. NMR nuclear magnetic resonance, ESI-MS electrospray ionization mass spectrometry, δH ^{1}H-NMR chemical shift, δC ^{13}C-NMR chemical shift, $CDCl_3$ deuterated chloroform

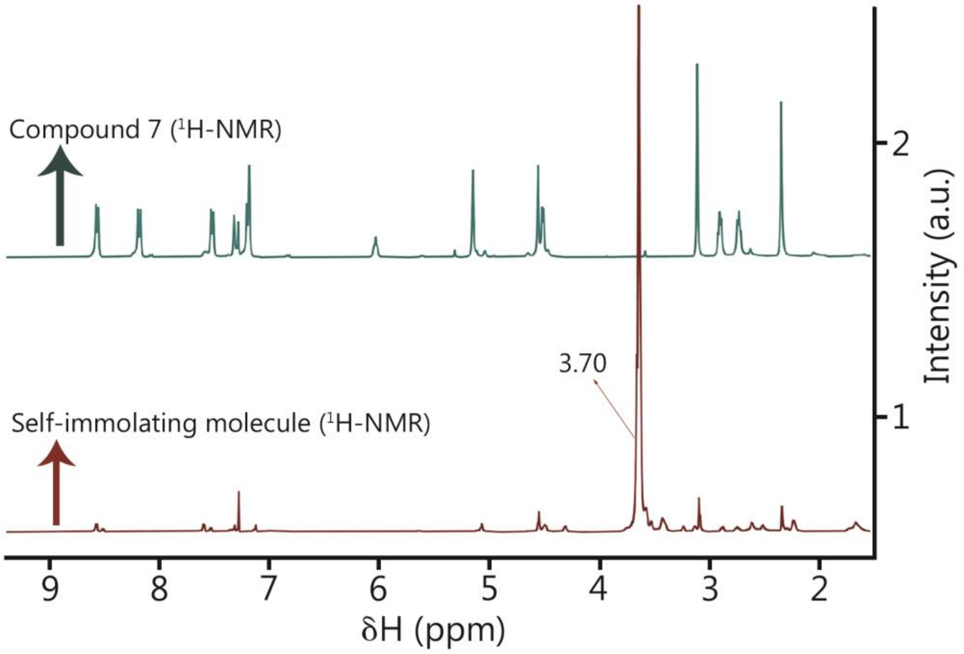

Fig. S11 ^{1}H-NMR spectrum of self-immolating spacer in methanol-*d4*. NMR nuclear magnetic resonance, δH ^{1}H-NMR chemical shift

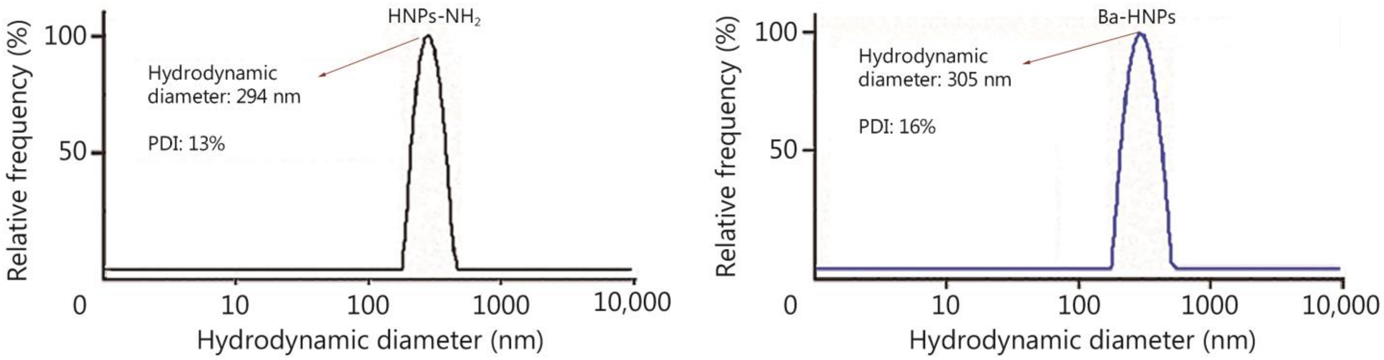

Fig. S12 DLS result of HNPs-NH_2 and Ba-HNPs. DLS dynamic light scattering, HNPs-NH_2 amino-modified hydrogel nanoparticles, Ba-HNPs Ba^{2+}-loaded hydrogel nanoparticles, PDI polydispersity index

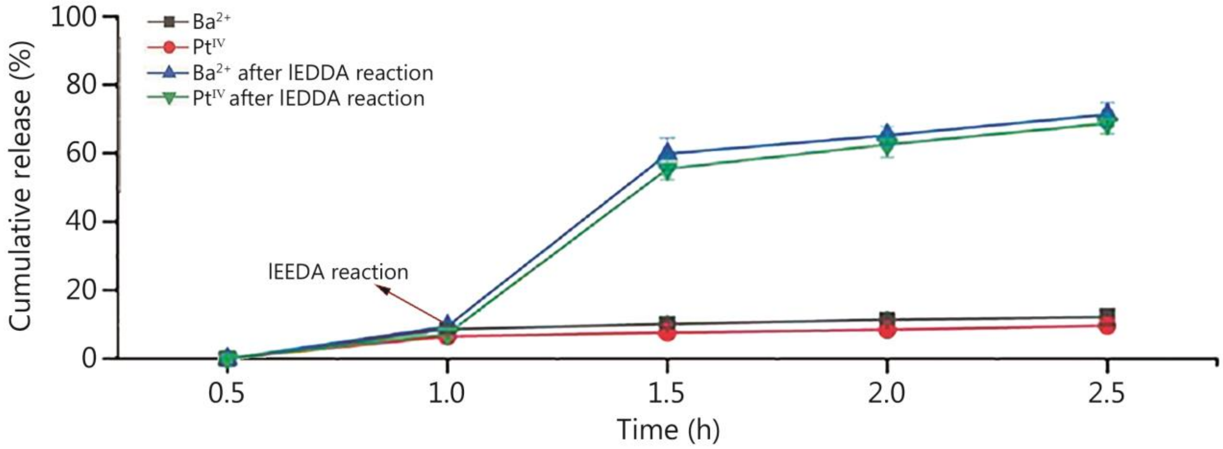

Fig. S13 The release of Ba^{2+} and Pt^{IV} from nanogel. Pt^{IV} platinum^{IV}, IEDDA inverse electron demand Diels-Alder

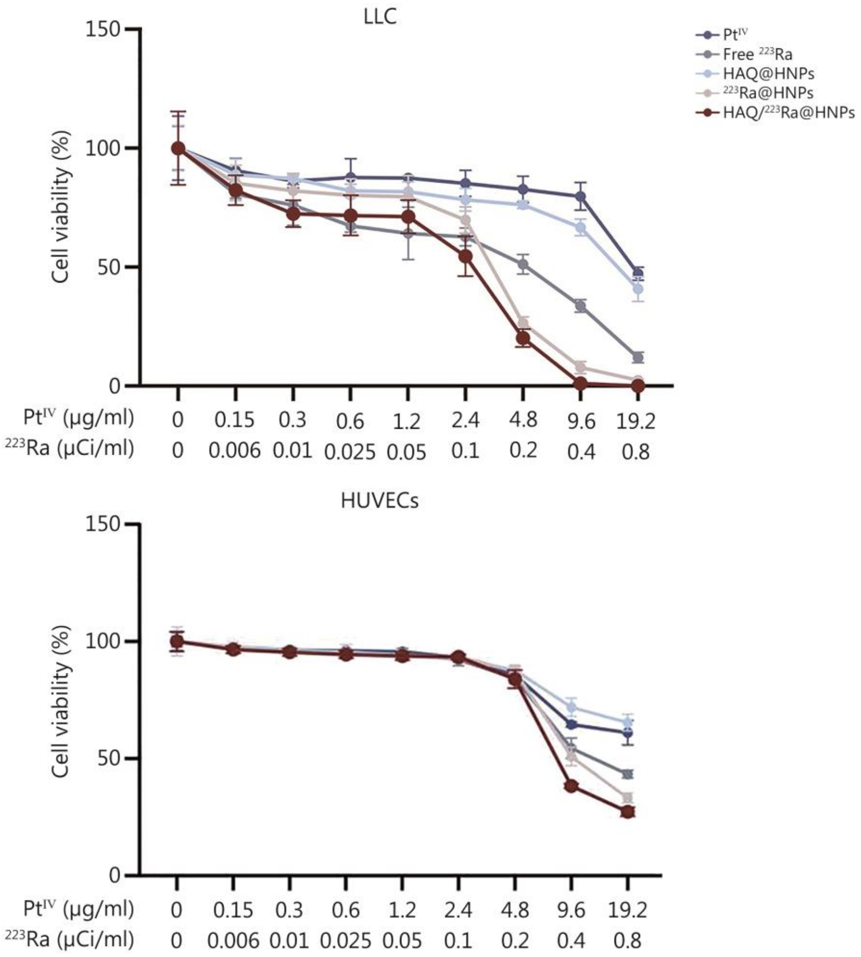

Fig. S14 Cytotoxicity of HAQ/^{223}Ra@HNPs in vitro. Relative viabilities of LLC cells and HUVECs under different treatments for 24 h (n = 4). LLC Lewis lung carcinoma, HUVECs human umbilical vein endothelial cells, Pt^{IV} platinum^{IV}, HAQ@HNPs platinum^{IV} (Pt^{IV})-loaded hydrogel nanoparticles with HAQ, ^{223}Ra@HNPs ^{223}Ra-loaded hydrogel nanoparticles with self-immolating molecule, HAQ/^{223}Ra@HNPs Pt^{IV}-loaded hydrogel nanoparticles with HAQ and ^{223}Ra-loaded hydrogel nanoparticles with self-immolating molecule

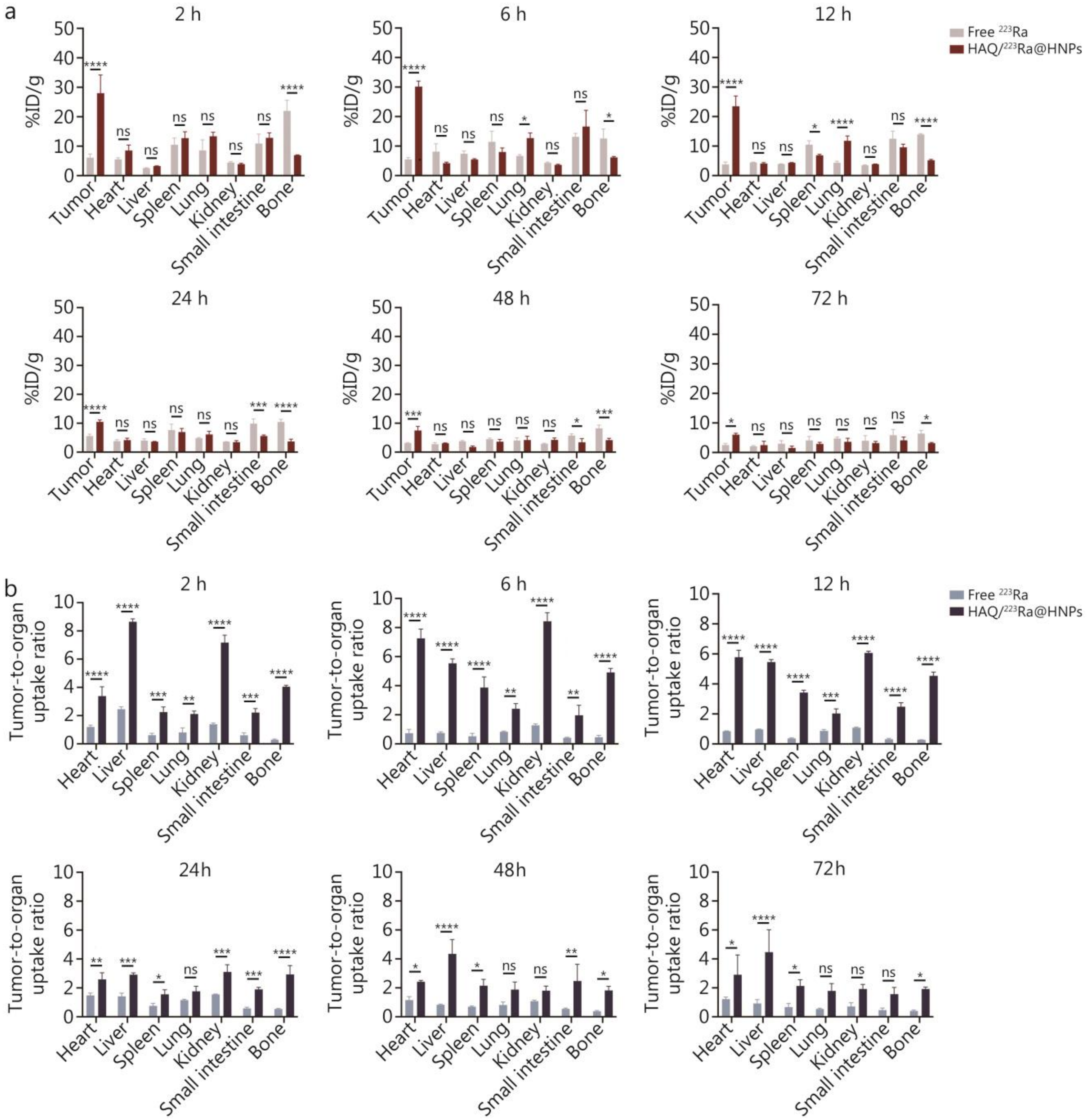

Fig. S15 Biodistribution profile. **a** The radioactivity concentrations of free ^{223}Ra and HAQ/^{223}Ra@HNPs in B16/F10-bear mice at 2, 6, 12, 24, 48, 72 h post injection (n = 3). **b** The tumor-to-organ uptake ratios of free ^{223}Ra and HAQ/^{223}Ra@HNPs in B16/F10-bearing mice at 2, 6, 12, 24, 48, 72 h post injection (n = 3). Data are expressed as mean ± SD. $^{*}P < 0.05$, $^{**}P < 0.01$, $^{***}P < 0.001$, $^{****}P < 0.0001$. SD standard deviation, HAQ/^{223}Ra@HNPs platinum^{IV} (Pt^{IV})-loaded hydrogel nanoparticles with HAQ and ^{223}Ra-loaded hydrogel nanoparticles with self-immolating molecule. ns non-significant

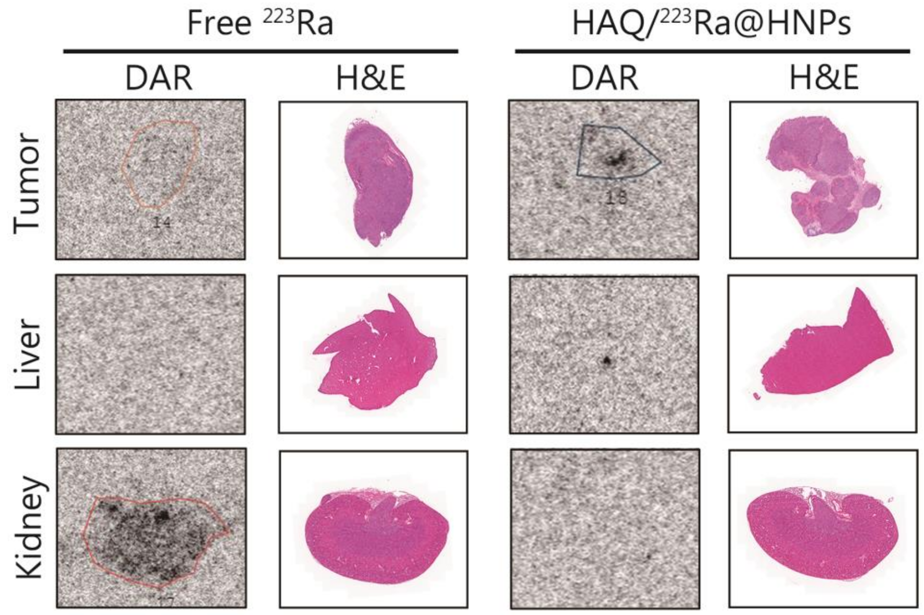

Fig. S16 Representative DAR and H&E images of frozen tissue sections from B16/F10-bearing mice following different treatments at 72 h post-injection. DAR digital autoradiography, HAQ/^{223}Ra@HNPs platinum^{IV} (Pt^{IV})-loaded hydrogel nanoparticles with HAQ and ^{223}Ra-loaded hydrogel nanoparticles with self-immolating molecule

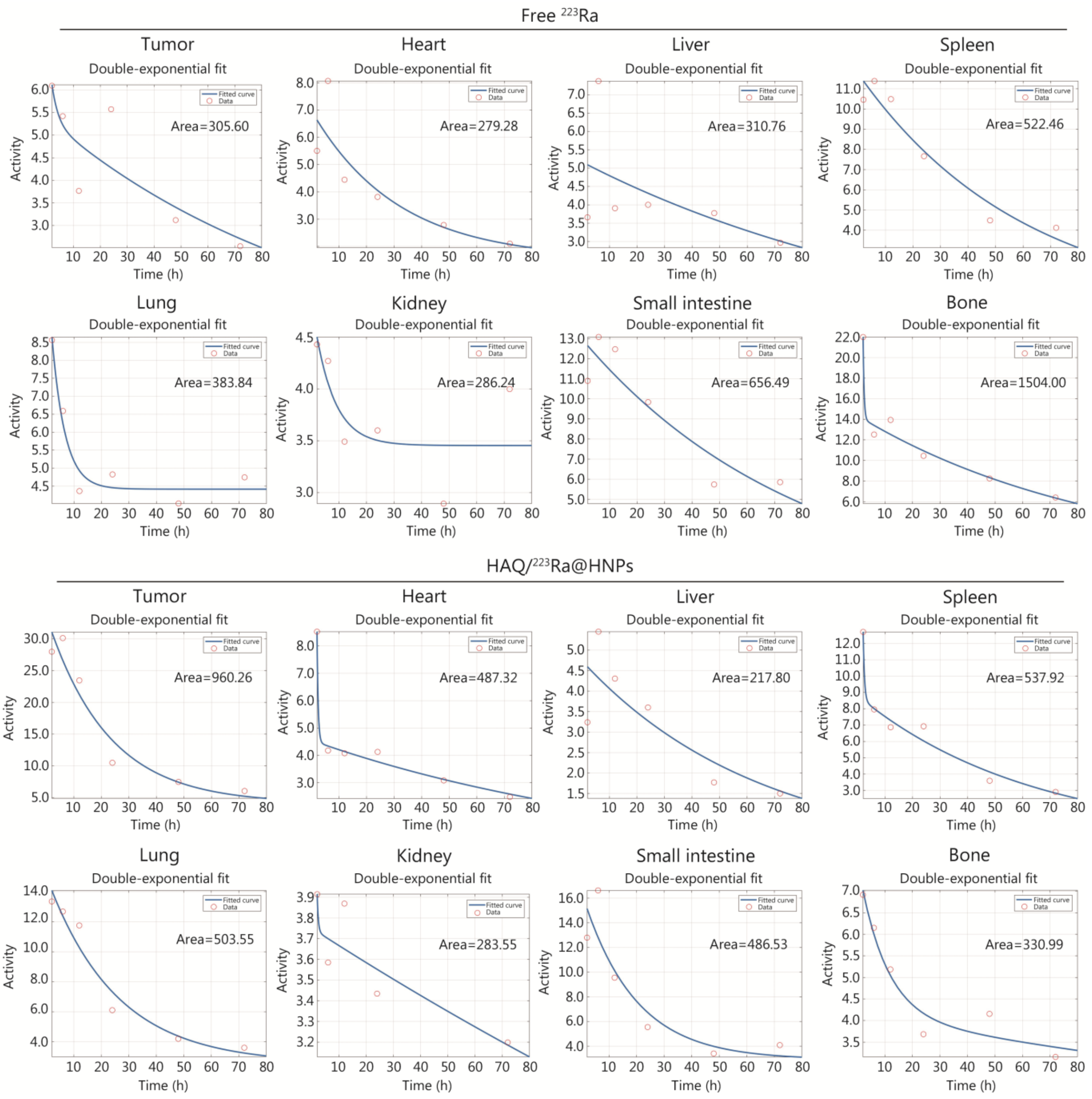

Fig. S17 Time-activity curves and fitted functions for biodistribution of ^{223}Ra and HAQ/^{223}Ra@HNPs. HAQ/^{223}Ra@HNPs platinum^{IV} (Pt^{IV})-loaded hydrogel nanoparticles with HAQ and ^{223}Ra-loaded hydrogel nanoparticles with self-immolating molecule

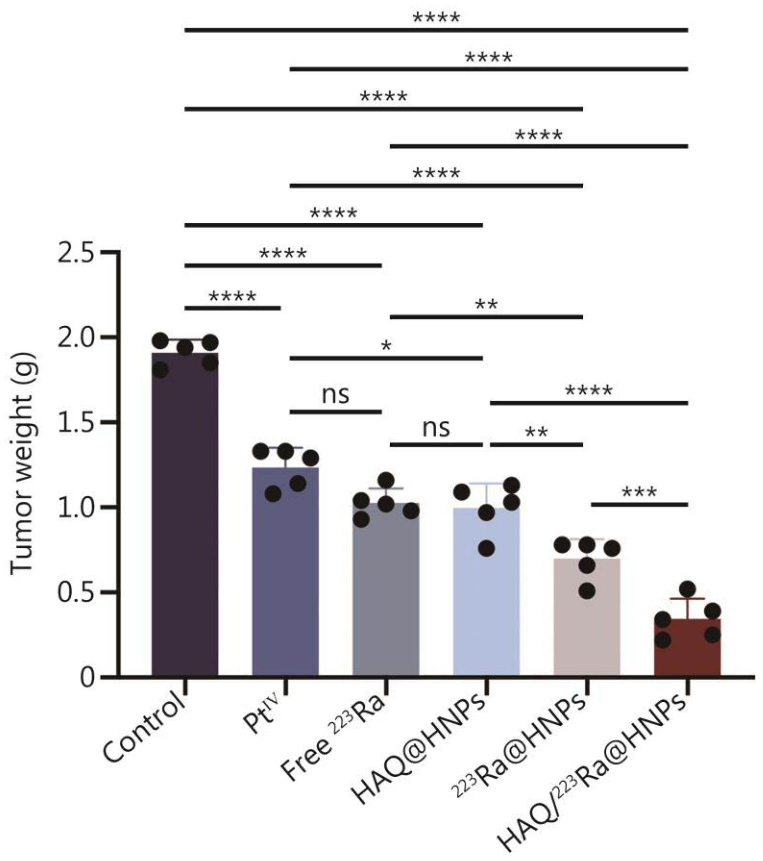

Fig. S18 Ex vivo tumor weights from each group at day 10 (n = 5) in B16/F10 tumor-bearing mice. Data are expressed as mean ± SD. $^{*}P < 0.05$, $^{**}P < 0.01$, $^{***}P < 0.001$, $^{****}P < 0.0001$. SD standard deviation, HAQ@HNPs platinum^{IV} (Pt^{IV})-loaded hydrogel nanoparticles with HAQ, ^{223}Ra@HNPs ^{223}Ra-loaded hydrogel nanoparticles with self-immolating molecule, HAQ/^{223}Ra@HNPs Pt^{IV}-loaded hydrogel nanoparticles with HAQ and ^{223}Ra-loaded hydrogel nanoparticles with self-immolating molecule, ns non-significant

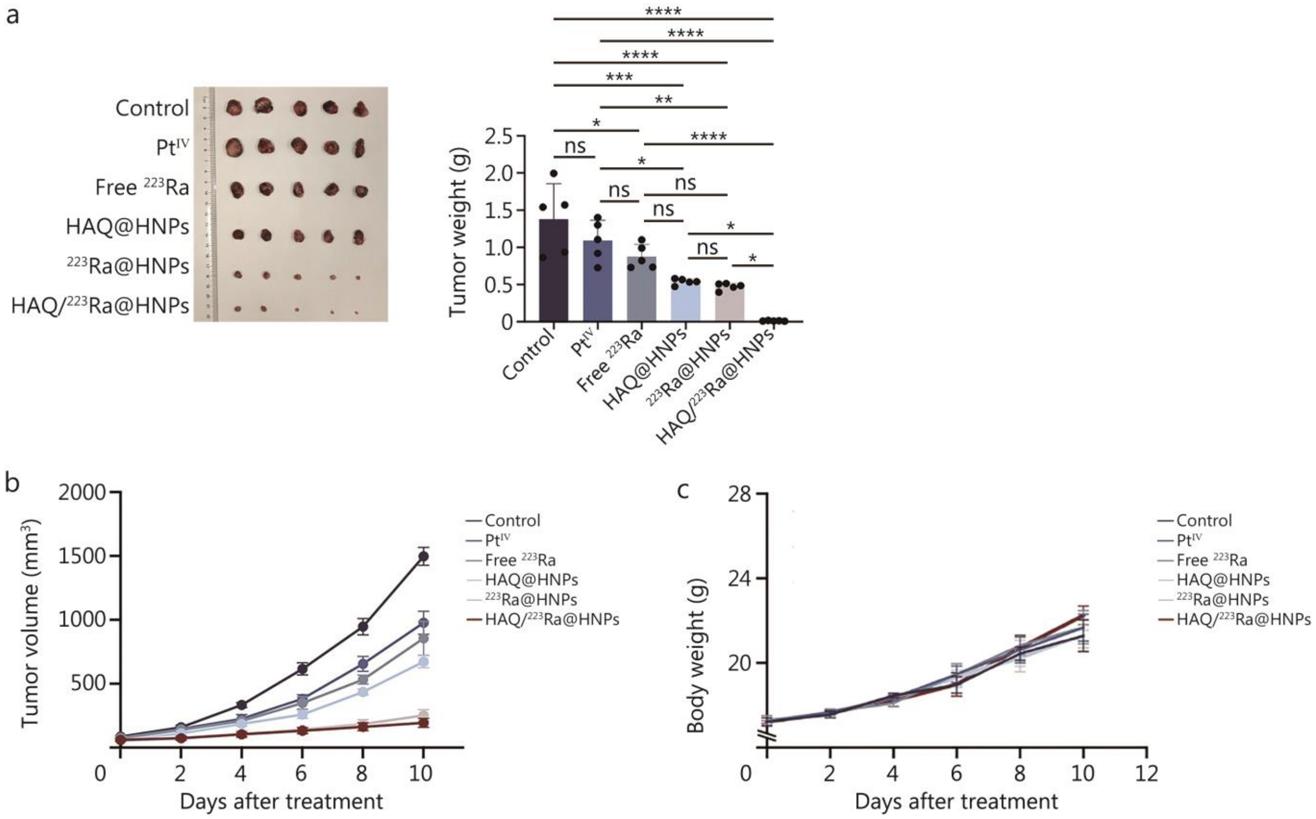

Fig. S19 Antitumor evaluations of HAQ/^{223}Ra@HNPs in LLC tumor-bearing mice. **a** Ex vivo tumor images and weights from each group at day 10 (n = 5). **b** Individual tumor growth curves of LLC tumor-bearing mice from different treatment groups (n = 5). **c** Body weight of mice after different treatments (n = 5). Data are expressed as mean ± SD. $^{*}P < 0.05$, $^{**}P < 0.01$, $^{***}P < 0.001$, $^{****}P < 0.0001$. SD standard deviation, Pt^{IV} platinum^{IV}, HAQ@HNPs platinum^{IV} (Pt^{IV})-loaded hydrogel nanoparticles with HAQ, ^{223}Ra@HNPs ^{223}Ra-loaded hydrogel nanoparticles with self-immolating molecule, HAQ/^{223}Ra@HNPs Pt^{IV}-loaded hydrogel nanoparticles with HAQ and ^{223}Ra-loaded hydrogel nanoparticles with self-immolating molecule, ns non-significant

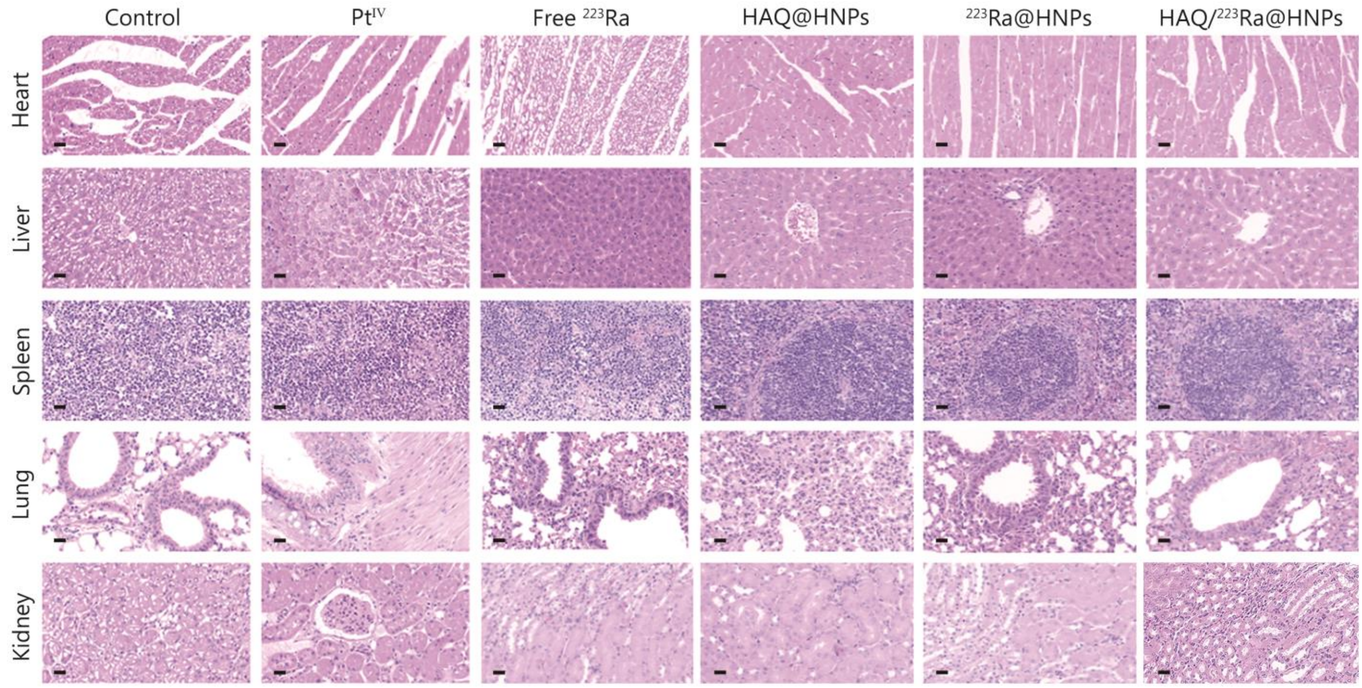

Fig. S20 Representative histological images of the main organs in B16/F10-bearing mice after different treatments, stained with hematoxylin and eosin. Scale bar = 20 μm. HAQ@HNPs platinum^{IV} (Pt^{IV})-loaded hydrogel nanoparticles with HAQ, ^{223}Ra@HNPs ^{223}Ra-loaded hydrogel nanoparticles with self-immolating molecule, HAQ/^{223}Ra@HNPs Pt^{IV}-loaded hydrogel nanoparticles with HAQ and ^{223}Ra-loaded hydrogel nanoparticles with self-immolating molecule

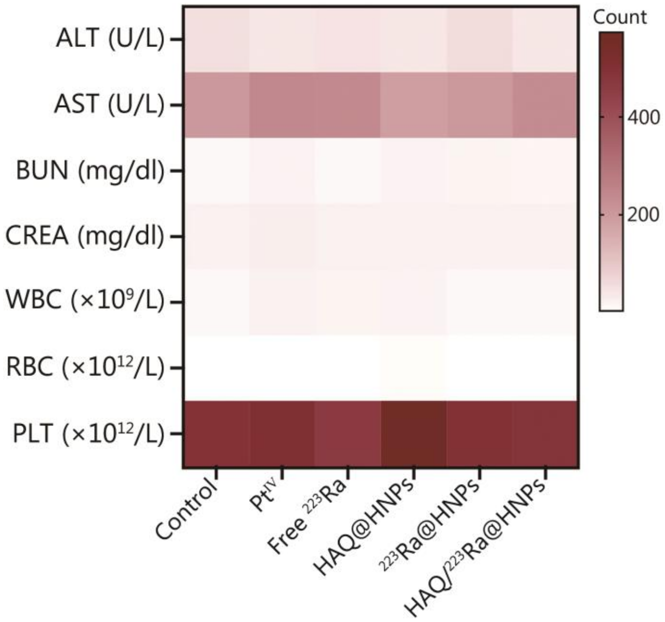

Fig. S21 Hematological analysis was performed on blood withdrawn from B16/F10 tumor-bearing mice in corresponding treatment groups at the terminal of study ($n = 5$). ALT alanine aminotransferase, AST aspartate aminotransferase, BUN blood urea nitrogen, CREA creatinine, WBC white blood cell, RBC red blood cell, PLT platelet, HAQ@HNPs platinum^{IV} (Pt^{IV})-loaded hydrogel nanoparticles with HAQ, ^{223}Ra@HNPs ^{223}Ra-loaded hydrogel nanoparticles with self-immolating molecule, HAQ/^{223}Ra@HNPs Pt^{IV}-loaded hydrogel nanoparticles with HAQ and ^{223}Ra-loaded hydrogel nanoparticles with self-immolating molecule

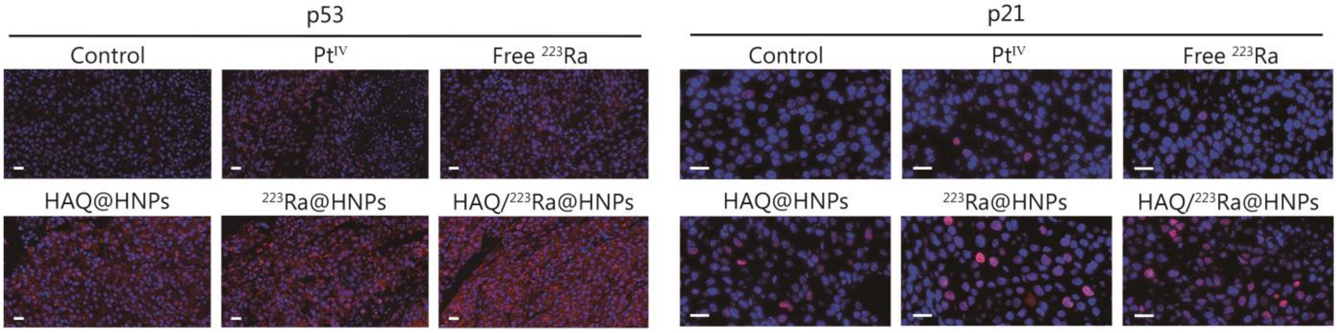

Fig. S22 Immunofluorescence staining of p53 and p21 in tumor tissues following different treatments. Scale bar = 20 μm. HAQ@HNPs platinum^{IV} (Pt^{IV})-loaded hydrogel nanoparticles with HAQ, ^{223}Ra@HNPs ^{223}Ra-loaded hydrogel nanoparticles with self-immolating molecule, HAQ/^{223}Ra@HNPs Pt^{IV}-loaded hydrogel nanoparticles with HAQ and ^{223}Ra-loaded hydrogel nanoparticles with self-immolating molecule

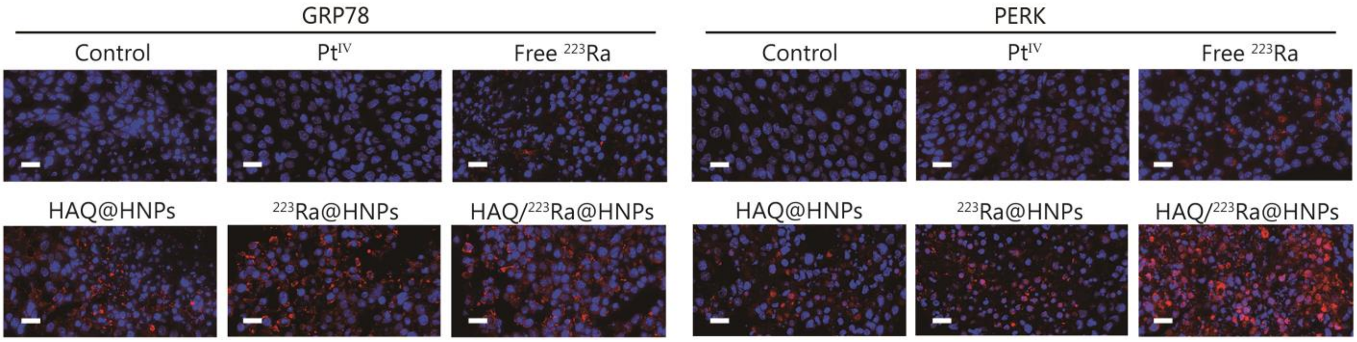

Fig. S23 Immunofluorescence staining of GRP78 and PERK in tumor tissues following different treatments. Scale bar = 20 μm. GRP78 glucose-regulated protein 78, PERK PKR-like endoplasmic reticulum kinase, HAQ@HNPs platinum^{IV} (Pt^{IV})-loaded hydrogel nanoparticles with HAQ, ^{223}Ra@HNPs ^{223}Ra-loaded hydrogel nanoparticles with self-immolating molecule, HAQ/^{223}Ra@HNPs Pt^{IV}-loaded hydrogel nanoparticles with HAQ and ^{223}Ra-loaded hydrogel nanoparticles with self-immolating molecule

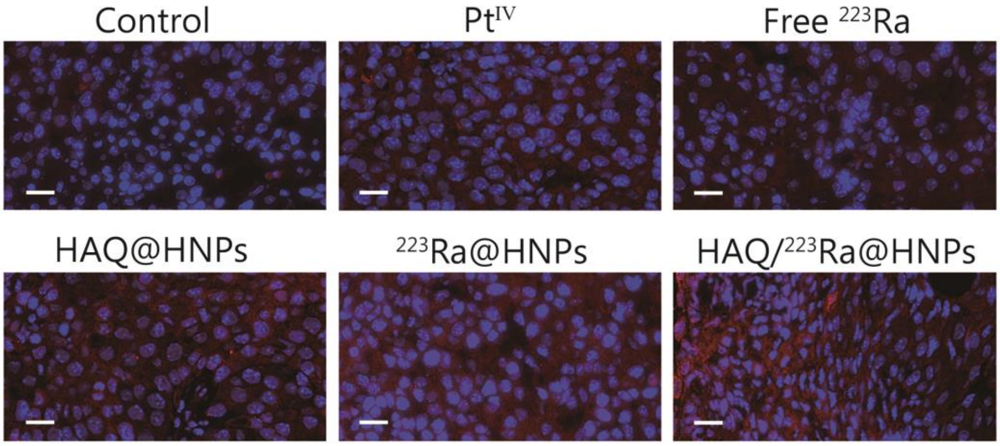

Fig. S24 Immunofluorescence staining of PD-L1 in tumor tissues following different treatments. Scale bar = 20 μm. HAQ@HNPs platinum^{IV} (Pt^{IV})-loaded hydrogel nanoparticles with HAQ, ^{223}Ra@HNPs ^{223}Ra-loaded hydrogel nanoparticles with self-immolating molecule, HAQ/^{223}Ra@HNPs Pt^{IV}-loaded hydrogel nanoparticles with HAQ and ^{223}Ra-loaded hydrogel nanoparticles with self-immolating molecule, PD-L1 programmed death-ligand 1

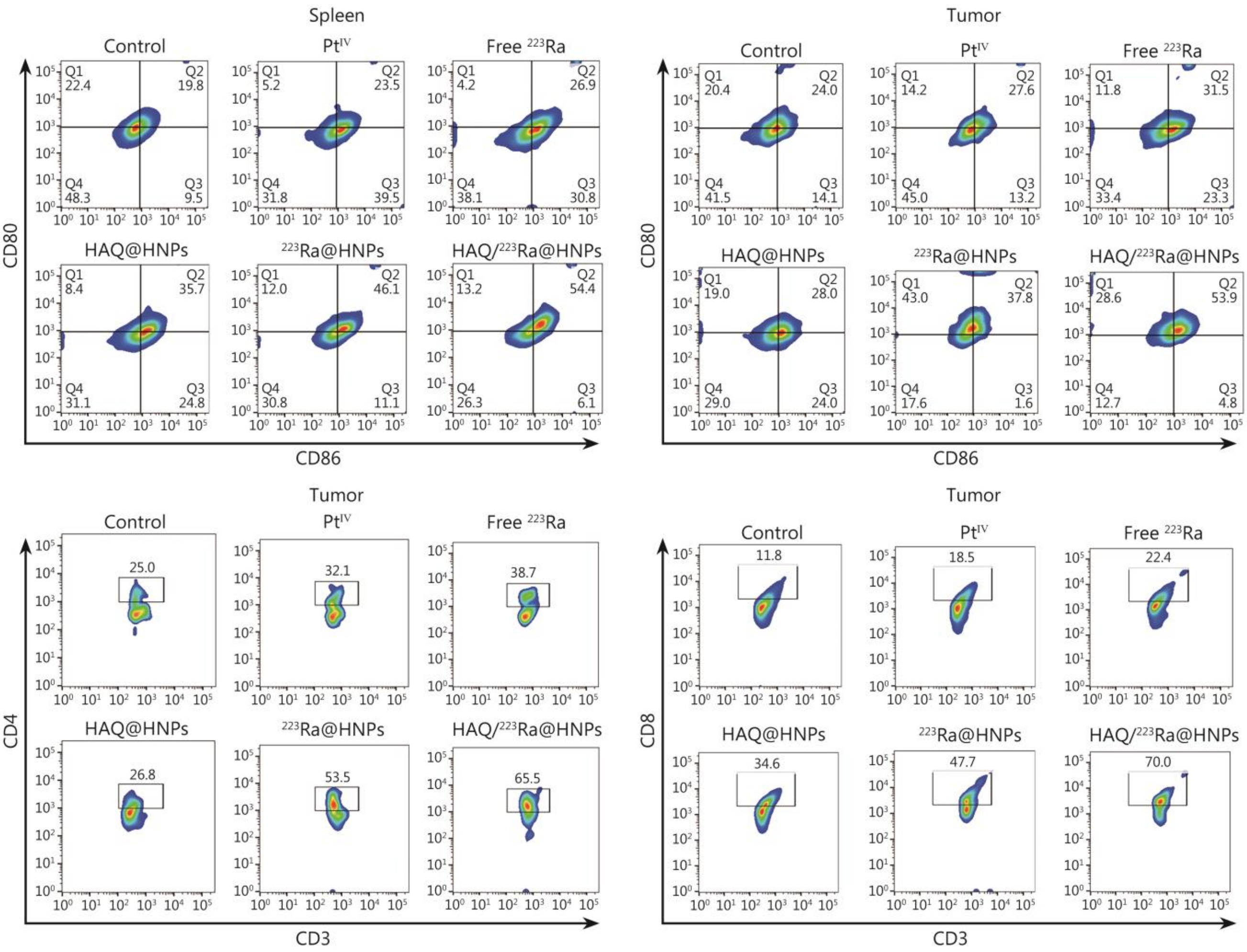

Fig. S25 Representative flow cytometry plots showing DCs maturation in the spleen, DCs maturation, $CD4^+$ T cells, and $CD8^+$ T cells in tumors from different groups (n = 3). HAQ@HNPs platinum^{IV} (Pt^{IV})-loaded hydrogel nanoparticles with HAQ, ^{223}Ra@HNPs ^{223}Ra-loaded hydrogel nanoparticles with self-immolating molecule, HAQ/^{223}Ra@HNPs Pt^{IV}-loaded hydrogel nanoparticles with HAQ and ^{223}Ra-loaded hydrogel nanoparticles with self-immolating molecule

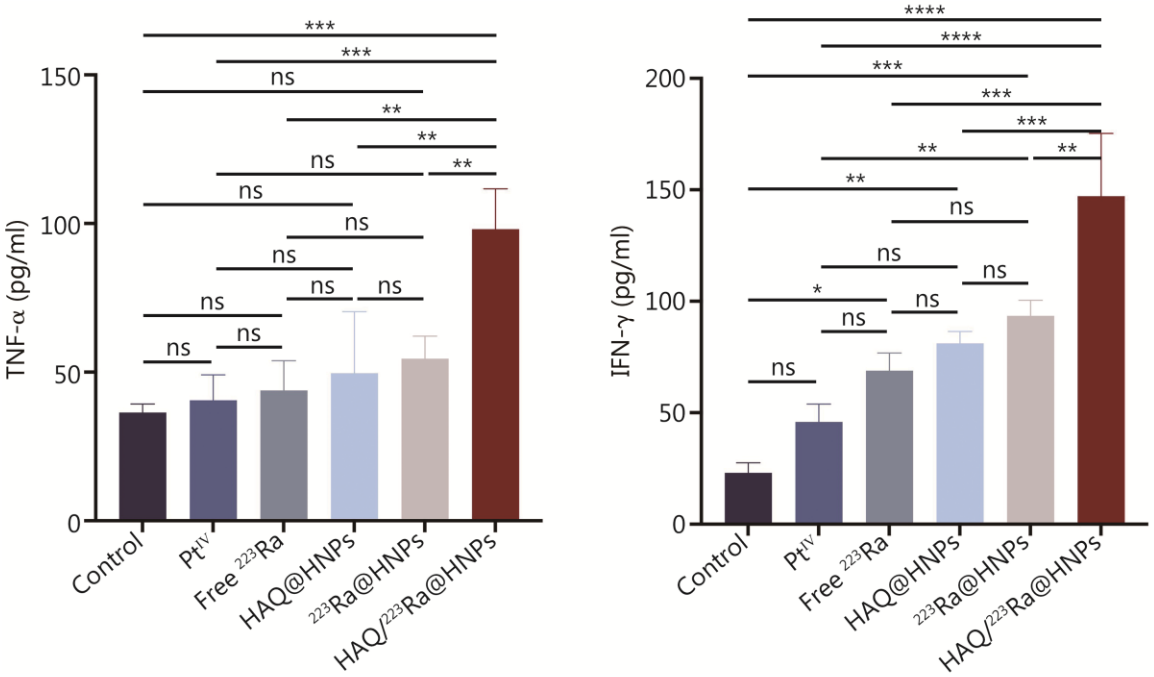

Fig. S26 The secretion of cytokines TNF-α and IFN-γ. Data are expressed as mean ± SD. $^{*}P < 0.05$, $^{**}P < 0.01$, $^{***}P < 0.001$, $^{****}P < 0.0001$, ns non-significant. TNF-α tumor necrosis factor-α, IFN-γ interferon-γ, Pt^{IV} platinum^{IV}, HAQ@HNPs Pt^{IV}-loaded hydrogel nanoparticles with HAQ, ^{223}Ra@HNPs ^{223}Ra-loaded hydrogel nanoparticles with self-immolating molecule, HAQ/^{223}Ra@HNPs Pt^{IV}-loaded hydrogel nanoparticles with HAQ and ^{223}Ra-loaded hydrogel nanoparticles with self-immolating molecule

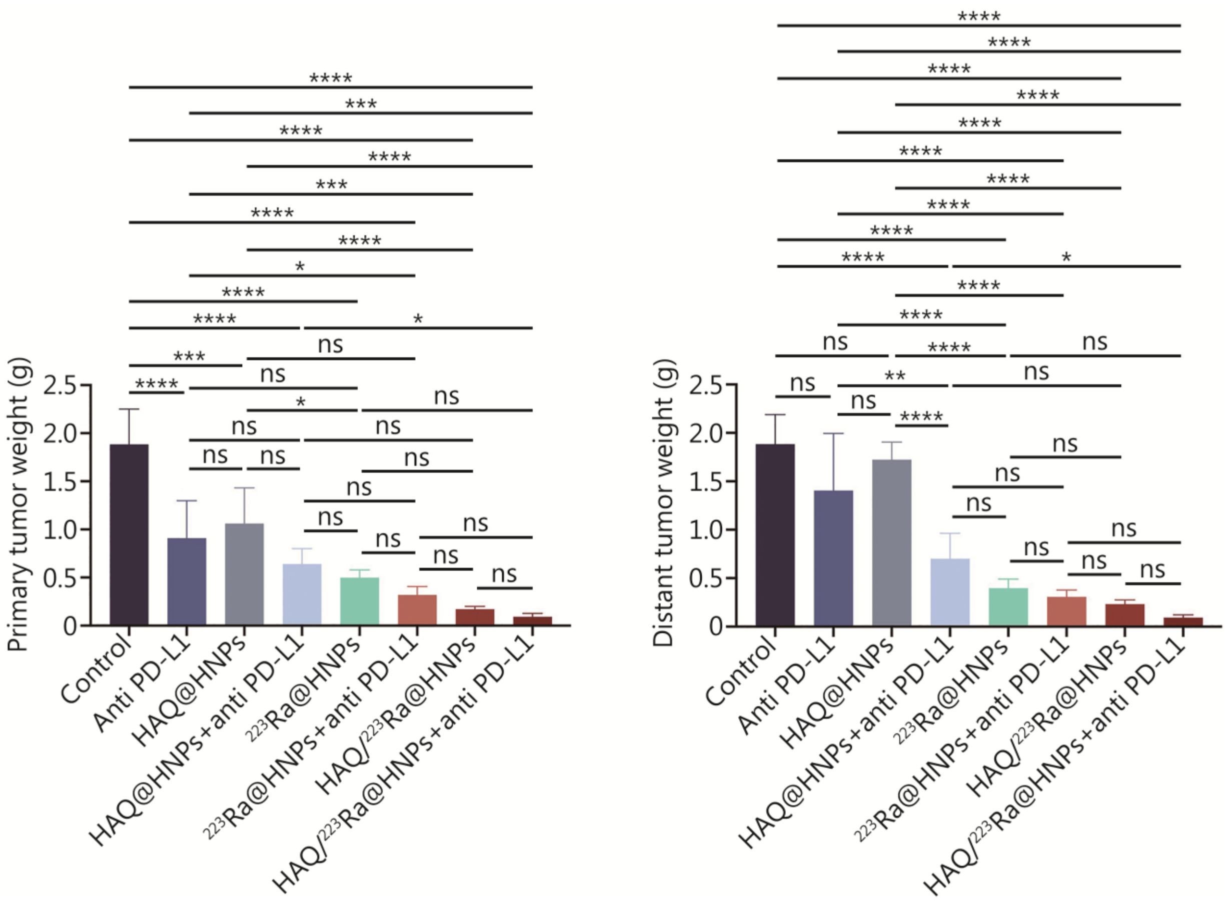

Fig. S27 Ex vivo tumor weight of excised primary and distant tumors from the mice after 16 d in corresponding groups (n = 5). $^{*}P < 0.05$, $^{**}P < 0.01$, $^{***}P < 0.001$, $^{****}P < 0.0001$. Pt^{IV} platinum^{IV}, HAQ@HNPs Pt^{IV}-loaded hydrogel nanoparticles with HAQ, ^{223}Ra@HNPs ^{223}Ra-loaded hydrogel nanoparticles with self-immolating molecule, HAQ/^{223}Ra@HNPs Pt^{IV}-loaded hydrogel nanoparticles with HAQ and ^{223}Ra-loaded hydrogel nanoparticles with self-immolating molecule, ns non-significant

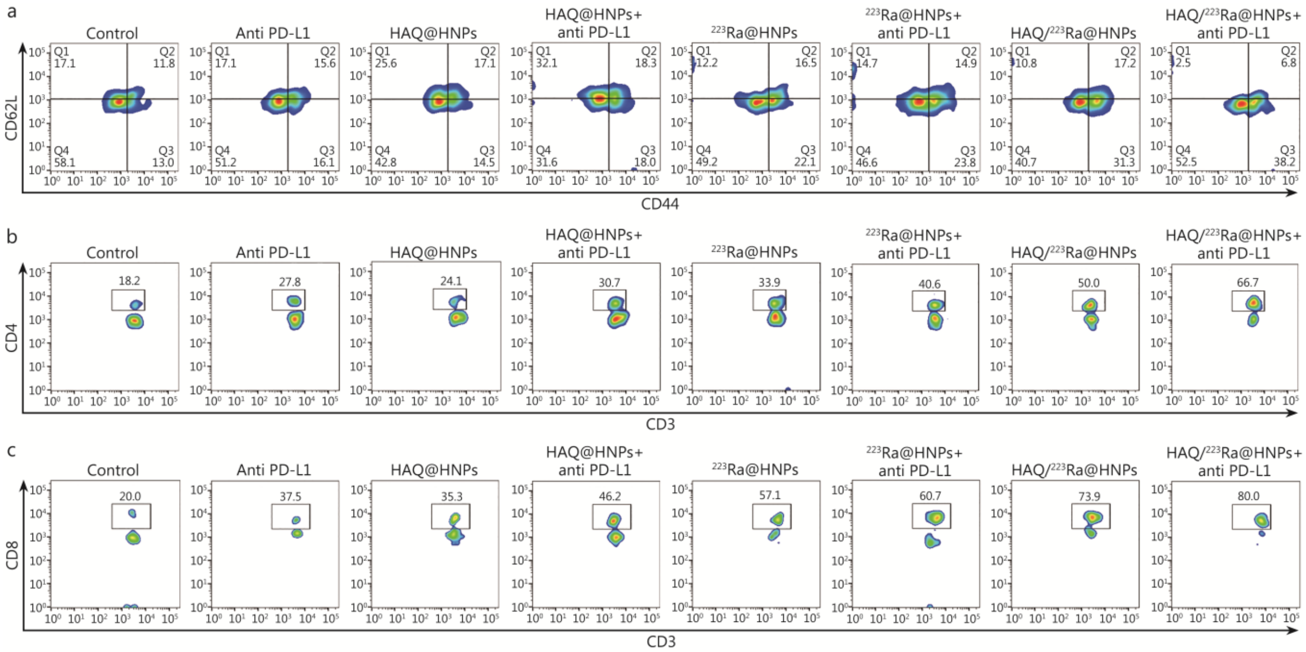

Fig. S28 Representative flow cytometry plots showing T_{EM} cells in spleen (**a**), $CD4^+$ T cells (**b**), and $CD8^+$ T cells (**c**) in distant tumors from different groups (n = 3). Pt^{IV} platinum^{IV}, HAQ@HNPs Pt^{IV}-loaded hydrogel nanoparticles with HAQ, ^{223}Ra@HNPs ^{223}Ra-loaded hydrogel nanoparticles with self-immolating molecule, HAQ/^{223}Ra@HNPs Pt^{IV}-loaded hydrogel nanoparticles with HAQ and ^{223}Ra-loaded hydrogel nanoparticles with self-immolating molecule

Table S1 Characteristics of the nanoparticles (mean ± SD)

Name	Hydrodynamic	Zeta potential (mV)	PDI	Loading
HNPs-COOH	283 ± 2	–6.32 ± 1.18	0.22 ± 0.02	/
Pt^{IV}@HNPs	300 ± 5	–14.81 ± 1.09	0.14 ± 0.01	Pt^{IV}
HAQ@HNPs	350 ± 12	–16.11 ± 1.10	0.27 ± 0.05	Pt^{IV}
HNPs-NH_2	294 ± 5	36.11 ± 1.12	0.13 ± 0.01	/
Ba-HNPs	305 ± 6	41.09 ± 1.00	0.16 ± 0.02	Ba^{2+}
Ba@HNPs	309 ± 4	33.51 ± 0.65	0.21 ± 0.05	Ba^{2+}

Pt^{IV} platinum^{IV}, *HAQ@HNPs* Pt^{IV}-loaded hydrogel nanoparticles with HAQ, *^{223}Ra@HNPs* ^{223}Ra-loaded hydrogel nanoparticles with self-immolating molecule, *HAQ/^{223}Ra@HNPs* Pt^{IV}-loaded hydrogel nanoparticles with HAQ and ^{223}Ra-loaded hydrogel nanoparticles with self-immolating molecule, *PDI* polydispersity index, "/" indicate no data

Table S2 Absorbed doses in each organ after administration of ^{223}Ra and HAQ/^{223}Ra@HNPs (Gy)

**Group**	**Tumor**	**Heart**	**Liver**	**Spleen**	**Lung**	**Kidney**	**Small intestine**	**Bone**
Free ^{223}Ra	73.1	66.8	74.3	125.0	91.8	68.5	157.0	360.0
HAQ/^{223}Ra@HNPs	230.0	117.0	52.1	129.0	120.0	67.8	116.0	79.2

HAQ/^{223}Ra@HNPs Pt^{IV}-loaded hydrogel nanoparticles with HAQ and ^{223}Ra-loaded hydrogel nanoparticles with self-immolating molecule, *PDI* polydispersity index
